# Mapping the Prevalence of COVID-19 Vaccine Acceptance at the Global and Regional Level: A Systematic Review and Meta-Analysis

**DOI:** 10.3390/vaccines10091488

**Published:** 2022-09-07

**Authors:** Erika Renzi, Valentina Baccolini, Giuseppe Migliara, Ciro Bellotta, Mariateresa Ceparano, Pierluigi Donia, Carolina Marzuillo, Corrado De Vito, Paolo Villari, Azzurra Massimi

**Affiliations:** 1 Department of Public Health and Infectious Diseases, Sapienza University of Rome, 00185 Rome, Italy; 2 National Hospital A.O.R.N. “Antonio Cardarelli”, 80131 Naples, Italy

**Keywords:** vaccination, vaccine hesitancy, COVID-19, systematic review, meta-analysis

## Abstract

Despite the availability of effective and safe vaccines, the acceptance of COVID-19 vaccination is suboptimal. In this meta-analysis we quantified the prevalence estimates of COVID-19 vaccine acceptance with a specific focus on worldwide geographical differences. We searched PubMed, Scopus, Web of Science and PsycInfo up to April 2021 (PROSPERO ID: CRD42021235328). Generalized random-effects linear models with a logit link were used to calculate the pooled estimated rate of vaccine acceptance at both the global and regional level. A meta-regression analysis was performed to assess the association between COVID-19 vaccine acceptance and various characteristics of the studies. Overall, 71 articles yielding 128 prevalence estimates were included. The pooled prevalence of COVID-19 vaccination acceptance rate was 66% (95% CI: 61–71%). This varied by geographic area, ranging from 36% (95% CI: 18–60%) in Africa to 83% (95% CI: 82–84%) in Oceania, and there was high variability between countries (15.4% Cameroon–100% Bhutan). Meta-regression analysis showed that studies that investigated COVID-19 vaccination intentions using multiple choice/scoring gave a vaccine acceptance prevalence lower than studies with only two possible answers (yes/no) (ß: −1.02 95% CI: −1.41 to −0.63). Despite some variation in the estimates, the results showed that one in three people may refuse/delay COVID-19 vaccination.

## 1. Introduction

Vaccines are one of the most effective tools for primary prevention of communicable diseases, including COVID-19 [1]. Vaccination directly protects vaccinated individuals and indirectly protects those who cannot be immunised through the development of community immunity [2]. However, to be truly successful in preventing and halting epidemics, immunization programs require high population coverage [3]. As of June 2022, global COVID-19 vaccine uptake is 66.4% [4], despite World Health Organization (WHO) requiring no less than 70% anti-SARS-CoV-2 immunization coverage by mid-2022 in all countries as an imperative [5]. To accelerate COVID-19 vaccine uptake, it is essential to identify the main barriers to vaccine acceptance. In the early stages of the immunization campaign, factors that reduced uptake were insufficient vaccine availability, problems with distribution and allocation of vaccines, and the organization of health services [6]. Today, 18 months after the start of the immunization campaign, with vaccine production and supply potentially adequate to meet global demand [7], it is clear that, alongside the logistical and access factors, individuals may remain unvaccinated for a variety of reasons, increasingly linked to individual attitudes and intentions towards vaccination [8]. These “hesitant” individuals represent a target group of particular interest to public health, since through the analysis of vaccination acceptance and its determinants, effective strategies can be implemented to fight the phenomenon of vaccine hesitancy [9,10].

Vaccine hesitancy is defined by the Strategic Advisory Group of Experts on Immunization (SAGE) as a “delay in acceptance or refusal of vaccination despite availability of vaccination services” [11]. This phenomenon, already identified by WHO in 2019 as one of ten threats to global health [12], also affects COVID-19 vaccination, resulting in suboptimal levels of vaccination acceptance and a failure to achieve community immunity [13]. Indeed, the scientific literature has reported significant variability in COVID-19 vaccine acceptance levels, with several countries reporting population vaccine intentions thresholds below 60% (Jordan, Cameroon, Russia, Poland, Croatia, and others) [14,15,16,17]. Monitoring trends in COVID-19 vaccine acceptance worldwide is critical when addressing vaccine hesitancy, especially where there is a need to develop tailored public health strategies that increase local vaccination uptake and promote a return to usual lifestyles and social activities [18].

Several systematic reviews and meta-analyses of COVID-19 vaccine intentions have already been conducted in the general population [19,20] and healthcare workers (HWs) [21,22], both before and after commercial release of authorized vaccines [23,24]. COVID-19 vaccine intentions have also been reviewed in other population subgroups [25,26]. However, to the best of our knowledge, data on the prevalence of anti-SARS-CoV-2 vaccination acceptance rates that focus on geographical differences are still lacking. Therefore, the primary objective of this review was to quantify estimates of COVID-19 vaccine acceptance before or in the very early stages of the vaccination campaign at both global and regional level. In addition, by analysing various characteristics of the studies, such as the target population investigated, when the survey was conducted, or study quality, we aimed to explore the variability in the prevalence estimates of COVID-19 vaccination acceptance.

## 2. Materials and Methods

This systematic review was performed according to the Cochrane Handbook for Systematic Reviews and the Preferred Reporting Items for Systematic Reviews and Meta-Analyses [27]. The review protocol was registered at PROSPERO (identifier CRD42021235328). Because this study did not involve primary data collection, the protocol was not submitted for institutional review board approval and did not require informed consent.

### 2.1. Search Strategy, Study Selection and Inclusion Criteria

The search was performed on PubMed, Scopus, Web of Science and PsycInfo, following database-specific search strategies on 3 April 2021. The main keywords used to search the database included “COVID-19”, “SARS-CoV-2”, “vaccine”, “vaccination”, “acceptance”, “intention”, “hesitancy”, “willingness”, and synonyms (the search strategy is fully reported in Supplementary Table S1). No restriction was applied. The search was supplemented by scanning the reference lists of the retrieved articles. Duplicate articles were removed, and the title and abstract of all retrieved records were screened. Studies that did not meet the inclusion criteria were excluded. Full texts of potentially relevant articles were examined by two researchers and reasons for exclusion were recorded. Any disagreement was resolved by discussion with a third author.

We included any study with the following characteristics: (i) reported in English or Italian, based on co-author language abilities; (ii) were peer-reviewed studies and had a cross-sectional design; (iii) investigated subjects aged ≥16 years (target population included in COVID-19 trials before vaccination release) (iv) provided data on earliest COVID-19 vaccine acceptance by the general population expressing their first vaccination intentions at the beginning of the vaccination campaign (March 2020–March 2021). We excluded records that investigated vaccination intentions in association with willingness to pay, or that focused on the theoretical efficacy of vaccinations in development, or from which data on the prevalence of COVID-19 vaccination intentions were not retrievable.

### 2.2. Data Collection and Quality Assessment

For each record included, two reviewers used a standardized data abstraction form to collect the following information: first author, year of publication, geographical area, country, target population, sample size, time of the investigation, general characteristics of the survey tool (structure of the questionnaire: domains, items, and data on validity of the instruments), number of answer options to the question on COVID-19 vaccination intention, and prevalence of COVID-19 vaccine acceptance (raw data or proportion, depending on data availability). Five geographical areas were considered: Africa, Asia, Europe, North America, Oceania, and South America. One survey [28] included participants from different geographical areas and was therefore considered separately. The target populations were classified as general population (i.e., without specific characteristics reported) and HWs. The period of investigation, which ranged from March 2020 to March 2021, was summarized into three categories based on the distribution of COVID-19 cases: March 2020–August 2020 (first wave period), September 2020–December 2020 (second wave period) and January 2021–March 2021 (COVID-19 vaccinations approved and administered). Sample size was deemed as small or large using the median value (i.e., 1052 participants) as cut-off. The number of answer options to the question on vaccine acceptance was classified into dichotomous (yes/no), more than two options, or not reported. Two independent reviewers performed a quality assessment of the studies included using the Newcastle-Ottawa Scale, which is designed to evaluate cross-sectional studies [29]. Any disagreement was resolved by discussion. Articles were considered of high quality when the total score was ≥7, fair quality if the score was ≥5 and <7, and poor quality if the score was less than 5.

### 2.3. Statistical Analysis

Since most articles provided two or more prevalence estimates (e.g., in different populations, in different countries), we considered each estimate separately. Generalized random-effects linear models with a logit link were used to calculate the pooled estimated proportions for vaccine acceptance [30], both overall and for each geographical area. The following stratification variables were considered: country, target population (general population and HWs), study quality (i.e., high quality when the total score was ≥7, fair/poor quality when the score was ≤6), sample size (small/large), period of investigation (March–August 2020, September-December 2020, January–March 2021), and answer options for the question on vaccine acceptance (dichotomous, more than two options, not reported). The *I*^2^ metric was used to test heterogeneity [31]. A random-effects meta-regression analysis using the restricted maximum-likelihood method and the Knapp-Hartung modification was performed to explore the association between study characteristics and the logit-transformed proportions of COVID-19 vaccination acceptance. We ran univariable and multivariable analyses including the covariates that could influence the pooled prevalence estimate based on literature review. The final model included the following variables: geographical area, target population, sample size, study quality, investigation period and number of answer options to COVID-19 vaccination intention. The lowest category was chosen as reference for study quality and sample size; for the target population and geographical area, we used the category with the highest number of studies; for the investigation period, we followed the calendar and used the first wave period, whereas for type of answer options we used the dichotomous category. In addition, separate meta-regression analyses were performed for geographical areas with more than 15 prevalence estimates. We used the same methods and variable selection process of the main analysis.

All calculations were performed using Stata (StataCorp LLC, 4905 Lakeway Drive, College Station, TX, USA), version 17.0. A two-sided *p*-value < 0.05 was considered statistically significant.

## 3. Results

### 3.1. Study Selection

**Figure 1:**
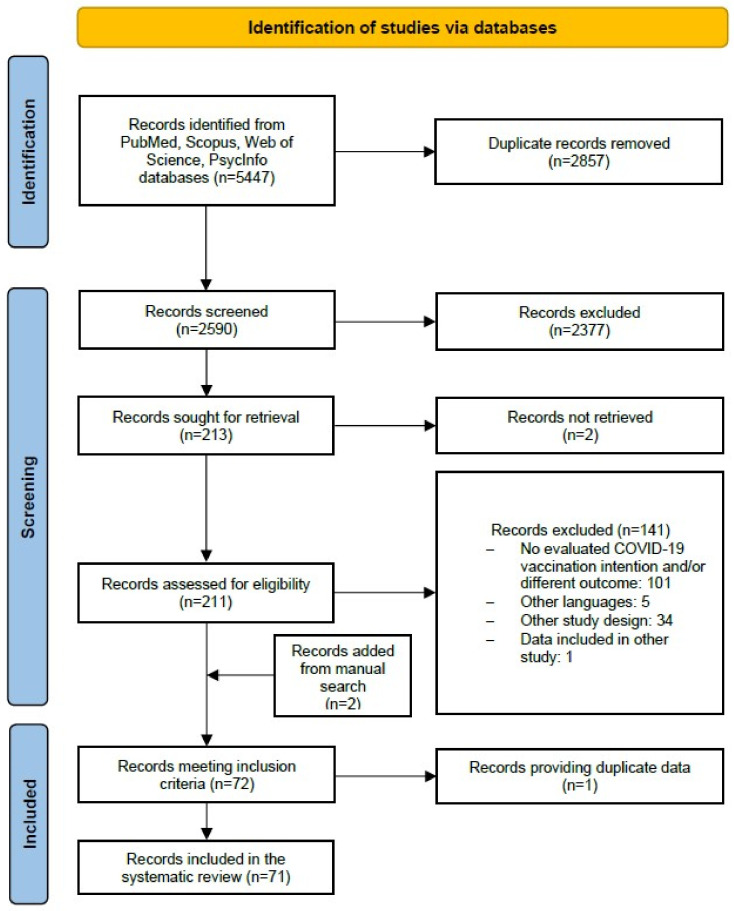
PRISMA flow diagram of the review process.

The systematic search yielded 5447 articles. After removal of duplicates, 2590 records were considered eligible (Figure 1). Screening by title and abstract returned 211 articles that were assessed by full-text analysis, of which 141 were excluded for the reasons outlined in Figure 1. Two records from a manual search were added to the previous 70, giving a total of 72 articles meeting the inclusion criteria. However, one of these duplicated data from another study and therefore was also excluded [14], leaving a total of 71 articles [10,15,16,17,28,32,33,34,35,36,37,38,39,40,41,42,43,44,45,46,47,48,49,50,51,52,53,54,55,56,57,58,59,60,61,62,63,64,65,66,67,68,69,70,71,72,73,74,75,76,77,78,79,80,81,82,83,84,85,86,87,88,89,90,91,92,93,94,95,96] that were ultimately included in the systematic review and that yielded 128 prevalence estimates of COVID-19 vaccination intention for meta-analysis.

### 3.2. Characteristics of the Prevalence Estimates Included in the Proportion Meta-Analysis

Most of the prevalence estimates came from Europe (N = 46), Asia (N = 31) and North America (N = 30), with 13 from South America, five from Africa, two from Oceania and one study that covered multiple countries simultaneously (Table 1). The most investigated countries were USA (N = 17), United Kingdom (N = 11) and Italy (N = 11). A total of 1,902,510 participants aged ≥16 years were included; of these, 46,252 were HWs. The sample size had a median value of 1052 individuals (range: 47–1,154,988). The investigation period most examined was March 2020–August 2020, during the first wave of the pandemic (N = 65; 50.8%), whereas 28 prevalence estimates (21.2%) related to COVID-19 vaccination intentions after the release of COVID-19 vaccines. As for the quality assessment, almost one in every four (31.3%) prevalence estimates were deemed of high quality, while the remaining 88 came from studies judged as being of fair or poor quality, mainly due to a lack of justification of sample size and/or comparability between responders and non-responders. The measurement tools used for data collection were mostly structured by the researchers based on the literature. The main features of the questionnaires were: (i) a range of 1–8 domains, mostly pertaining to the following areas of investigation: socio-demographic characteristics (e.g., sex, age, country, education, job position) knowledge (e.g., COVID-19 transmission, vaccination doses, administration), behavior and attitudes (e.g., adherence to COVID-19 containment measures, vaccination intentions) related to SARS-CoV-2 and COVID-19 vaccinations; and (ii) an average of 25 items (range 3–100). Only eight studies included a validated questionnaire designed specifically for COVID-19 vaccination [17,33,38,39,48,49,68,96]. COVID-19 vaccine intentions were investigated in 64% (82 investigations) of surveys with questions that allowed a choice between more than two answer options (e.g., score, scale, rating). Only 40 investigations provided just two answer options (Yes/No) to the question on COVID-19 vaccine intentions (e.g., Will you get vaccinated against COVID-19? (Yes/No)).

### 3.3. Proportion Meta-Analysis of COVID-19 Vaccination Intentions at the Global Level

**Figure 2:**
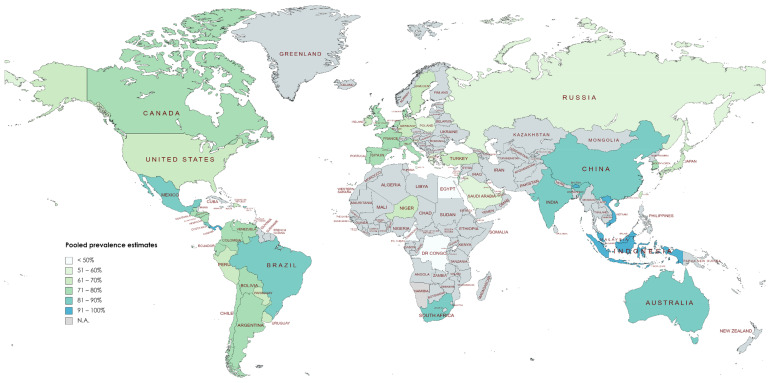
World map of pooled prevalence estimates of COVID-19 vaccination acceptance by country. NA: Not available.

Overall, the global pooled prevalence of COVID-19 vaccine acceptance was 66% (95% CI: 61–71%). Vaccination intentions varied markedly by country (Figure 2, Supplementary Figure S1). Heterogeneity was very high: in almost all cases, I2 was >95%. African countries reported the lowest levels of COVID-19 vaccination intention, with Cameroon, 15%, (95% CI: 14–17%), Democratic Republic of Congo, 28% (95% CI: 24–31%) and Egypt, 35% (95% CI: 33–37%) among the lowest, whereas Asian countries showed the highest level of acceptance, with rates above 80% in China (80%, 95% CI: 57–94%), India (82%, 95% CI: 80–84%), Indonesia (96%, 95% CI: 93–97%) and Bhutan (100%, 95% CI: 92–100%). However, lower estimates were reported in a few countries in the Middle East, namely United Arab Emirates, 54% (95% CI: 51–57%) and Saudi Arabia, 55% (95% CI: 46–63%). In Europe, most countries had pooled estimates ranging from 65% to 75% apart from a few exceptions, namely Slovenia, 56% (95% CI: 55–57%), Poland, 56% (95% CI: 52–60%) and Greece, 58% (95% CI: 55–61%). Malta had the lowest acceptance rate (50%, 95% CI: 48–52%, I2 = 28.4%), whereas Denmark had the highest (80%, 95% CI: 77–82%). North American prevalence estimates were heterogeneous, with vaccine acceptance levels higher than 80% in Mexico and lower than 70% in the United States. Finally, two South American countries had vaccination acceptance levels of 85%, Brazil (85%, 95% CI: 85–85%) and Costa Rica (85%, 95% CI: 84–86%), but in the other countries in this region the estimates ranged between 66% in Uruguay (95% CI: 65–67%) and 69% in Venezuela (95% CI: 68–70%).

### 3.4. Proportion Meta-Analysis of COVID-19 Vaccination Intention at the Regional Level

The proportion meta-analyses of COVID-19 vaccination intention at the regional level were stratified by target population, period of survey administration, study quality, type of answer, and sample size (Table 2).

#### 3.4.1. Africa

Five investigations with a total sample size of 6547 individuals were conducted in Africa (Table 2). Of these, three surveys were on general population, yielding a pooled prevalence of 41%, (95% CI: 10–82%), and two on HWs, accounting for a pooled prevalence of 33% (95% CI: 32–35%) (Supplementary Figure S2). Four investigations were collected in March 2020–August 2020, with a pooled prevalence of 37% (95% CI: 14–68%) and only one survey was conducted after the vaccination release (35%, 95% CI: 33–37%). Similar gaps in the pooled prevalence rates were found for study quality (high quality: 25% [95% CI: 17–36%]; and poor/fairy quality: 73% [95% CI: 71–75%], respectively), and type of answer options (multiple choices: 28% [95% CI: 24–31%]; two options: 73% [95% CI: 71–75%]; not specified: 24% [95% CI: 23–26%], respectively). Finally, the two surveys with a sample size >1052 participants gathered a lower vaccination intention compared to the other (<1052) (24% [95% CI: 23–26%] vs. 51% [95% CI: 21–80%]).

#### 3.4.2. Asia

Thirty-one investigations were conducted in Asia, involving a total of 40,656 individuals. The overall pooled prevalence estimate of vaccination acceptance was 66% (95% CI: 54–76%) (Table 2). Nineteen surveys investigated COVID-19 vaccination acceptance in the general population, with a pooled prevalence of 63% (95% CI: 50–74%), while 12 surveys were conducted on HWs, with a pooled prevalence of 70% (95% CI: 49–85%) (Supplementary Figure S2). Differences in vaccination acceptance levels were recorded according to the survey period (March–August 2020: 67% [95% CI: 51–80%]; September–December 2021: 74% [95% CI: 49–90%]; January–March 2021: 49% [95% CI: 31–67%]; and not specified: 50% [95% CI: 48–52%]). Providing more answer options in the COVID-19 vaccination intention assessment led to a lower pooled prevalence of vaccination acceptance compared to surveys with only two choices (55% [95% CI: 46–63%] vs. 84% [95% CI: 66–94%], respectively). By contrast, similar rates of vaccination intention were found in relation to the study quality and sample size (from 63% [95% CI: 46–78%] to 68% [95% CI: 51–78%] for the first, and from 64% [95% CI: 44–80%] to 67% [95% CI: 53–79%] for the latter).

#### 3.4.3. Europe

We included forty-six investigations in Europe involving 1,223,397 individuals, with a pooled prevalence estimate of COVID-19 vaccination acceptance of 71% (95% CI: 65–76%) (Table 2). The general population was investigated in 41 surveys with a pooled prevalence estimate of 71% (95% CI: 64–77%), while HWs yielded a pooled prevalence of 65% (95% CI: 55–74%) (Supplementary Figure S2). We found changes in the pooled prevalence estimate of COVID-19 vaccination acceptance across time periods (73% [95% CI: 66–79%] in March–August 2020; 69% [95% CI: 58–78%] in September–December 2021; 79% [95% CI: 75–83%] in January–March 2021; and 56% [95% CI: 43–68%] when not specified). Lastly, studies that collected data on COVID-19 vaccinations using questions characterized by dichotomous answers showed a pooled prevalence of 82% (95% CI: 74–88%), while studies that use multiple choice and/or scoring returned a vaccine acceptance of 67% (95% CI: 60–73%). In only one case was this not specified (86%, 95% CI: 83–89%). As for the other characteristics, similar rates of vaccination intention were found for study quality (from 68% [95% CI: 57–77%] to 73% [95% CI: 66–79%]) and sample size (from 69% [95% CI: 61–76%] to 73% [95% CI: 65–80%]).

#### 3.4.4. South America

Thirteen investigations were included for South America, all of them conducted on the general population (245,296 individuals) and accounting for an overall pooled estimate of COVID-19 vaccination acceptance of 81% (95% CI: 78–84%) (Table 2) (Supplementary Figure S2). Three investigations were conducted between March and August 2020, with a pooled prevalence estimate of COVID-19 vaccination acceptance of 82% (95% CI: 77–86%), a value in line with those surveys conducted in January–March 2021, which yielded a pooled prevalence of 80% (95% CI: 80–80%). Small differences between the pooled estimates were observed according to sample size (80% [95% CI: 80–80%] vs. 82% [95% CI: 77–86%]), type of answer options (79% [95% CI: 76–81%] vs. 82% [95% CI: 79–86%]) and study quality (85% [95% CI: 83–87%] vs. 80% [95% CI: 77–86%]).

#### 3.4.5. Oceania

Only two pooled prevalence were available for Oceania, yielding a pooled prevalence estimate of COVID-19 vaccination intention of 83% (95% CI: 82–84%).

### 3.5. Meta-Regression Analyses

The results of the meta-regression analyses are shown in Table 3. Africa was the only geographical area to have lower rates of acceptance than Europe (ß: −0.97, 95% CI: −1.38 to −0.11). In addition, compared to studies that quantified vaccine intentions through yes or no answers, surveys that offered multiple options (ß: −1.02 95% CI: −1.41 to −0.63) or did not specify the method used (ß: −1.37 95% CI: −2.15 to −0.60) had lower levels of COVID-19 vaccine acceptance. By contrast, no statistical difference was shown for target population, time of investigation, study quality and sample size.

As for the separate meta-regression analyses by geographical area, findings for Europe and Asia confirmed that offering multiple answer options compared to yes or no only in the vaccination intention question gave lower vaccination acceptance rates (ß: −0.82, 95% CI: −1.44 to −0.20 and ß: −2.11, 95% CI: −3.44 to −0.79, respectively), while in North America it led to a higher pooled estimate (ß: 1.16, 95% CI: 0.26 to 2.06) (Supplementary Table S2). Furthermore, vaccination intention in North America varied depending on the time of investigation, with estimates collected January 2021–March 2021 having higher acceptance rates than March-August 2020 (ß: 1.36, 95% CI: 0.47 to 2.25); there was also a borderline difference in relation to the sample size (ß: 0.51, 95% CI: −0.01 to 1.02). No other variables seemed to impact the results.

## 4. Discussion

Our systematic review analysed COVID-19 vaccination intentions up to March 2021 in 59 countries around the world and found that, although overall 66% of the general population was willing to accept the vaccination, there was considerable variation across different geographic areas. This is in line with real-life data showing that, as of June 2022, global vaccination coverage is around 66.4% [4] (defined as the population who have received at least one dose of a COVID-19 vaccine); this is below the 70% WHO-defined target to be reached by June 2022 [5].

At the regional level, the pooled estimates of vaccine acceptance were particularly low in Africa (36%), where current vaccination coverage amounts to approximately 20% of the population [97]. However, some caution must be shown in interpreting these results because of the small number of studies carried out in African countries, particularly given that the study of vaccine intention and hesitancy still appears to be a poorly understood and investigated phenomenon. A possible explanation for low levels of COVID-19 vaccine acceptance could be the presence of barriers relating to the availability and distribution of COVID-19 vaccines [98]. In fact, as reported by the Organization for Economic Cooperation and Development (OECD) in March 2021 [99], only four African countries had initiated administration of the COVID-19 vaccine by that time; similarly, geographic differences in vaccine availability persisted over time, while Australia (83% of pooled estimates of vaccine acceptance) had defined vaccination as a priority in investments, African countries continued to receive inadequate doses of vaccines. These issues have been partly addressed following the implementation of dedicated programs to ensure globally equitable access to vaccinations, such as the WHO-funded COVAX program [100]. Alongside the already mentioned contextual determinants and more generalized difficulties in accessing health and vaccination services, African countries seem to have both a lower perception of the risk of spreading COVID-19 infection and low levels of health literacy [101], demonstrating the need to enhance population engagement strategies [102,103,104] and to improve awareness of the risks associated [105] with vaccine hesitancy. However, some caution must be shown in interpreting these results because of the small number of studies carried out in African countries, particularly given that the study of vaccine intention and hesitancy still appears to be a poorly understood and investigated phenomenon. Most studies included in the meta-analysis were instead carried out in countries such as the USA, UK and Italy, where vaccine hesitancy has been thoroughly investigated over the years; there is therefore a need to increase research into vaccine hesitancy in lower- and middle-income countries [106] and to encourage tailored public-health strategies that address vaccine hesitancy.

As described above, we wanted to investigate whether vaccine acceptance estimates also depended on the population under investigation, with particular attention to HWs due to their central role in the fight against the pandemic. HWs are the most trusted figures when it comes to health advice and correct information on vaccines in their communities [107]. This role makes them responsible not only for their own vaccination choices, but also for engagement with the community over such decisions. Therefore, we would hypothesise that high levels of COVID-19 vaccine acceptance are expected from HWs. In contrast, the results of our meta-regression analyses did not show significant differences in COVID-19 vaccination acceptance between the general population and healthcare professionals. Indeed, despite their role, HWs demonstrated instead a tendency to lower vaccination acceptance estimates than the general population, as showed also by the meta-analyses of intention rates stratified by geographical area, with the exception of Asia. It should be pointed out that the search strategy used in this meta-analysis was not designed to uncover studies specifically and exclusively aimed at HWs, as the inclusion criteria generally targeted adults over the age of 16. Data for the target population “HWs” were extracted from the included surveys when reported by the authors. The current situation in several countries that have introduced mandatory vaccination for HWs, shown by recent systematic reviews [22,108,109] and our own findings, highlights the fact that HW hesitancy towards COVID-19 vaccination continues to be a public health problem, in line with pre-pandemic studies on HW hesitancy towards other recommended vaccines [110,111,112,113,114,115]. The main reasons for vaccine hesitancy in HWs could be their exposure to conflicting information on vaccine effectiveness and safety during clinical practice [62,116], and a lack of knowledge on how to interpret scientific evidence and identify reliable information sources [117,118,119]. Accordingly, it is essential that strategies be employed to increase the acceptance of vaccination among HWs by improving knowledge of scientific methodology and analysis of evidence. This might be achieved by university-level training and by implementing communication strategies aimed at health professionals [120,121].

A further driver of different levels of vaccine acceptance could be the particular phase of the pandemic during which the surveys were carried out [13]. It seems likely that specific elements of the different phases of the pandemic, including which variants of the virus are in circulation, mortality rates and disease severity, and even the strength of restrictive measures, could underpin a change in perception of the risk-benefit ratio, one of the well-known individual determinants of vaccination hesitancy [8], which also causes variation in vaccine intentions [122,123]. Likewise, effective authorization of the use of vaccines could change vaccine acceptance levels during vaccine trial phases, especially where perceived efficacy and safety of the vaccine are concerned [108,122,124,125,126,127,128]. However, in line with current literature, our meta-regression analysis showed no difference in vaccine acceptance levels between different phases of the pandemic (first and second wave), and before and after the release of COVID-19 vaccines [20]. Although historically the phenomenon of vaccine hesitancy appears to be strongly related to vaccine features and timing, no significant differences emerge in this study. This could be explained by the fact that, despite the different waves described by the epidemiological data, the studies included in this meta-analysis were all performed during an emergency phase (March 2020–March 2021) when perceptions of the pandemic remained almost stable [129]. Finally, it is interesting that the stratified meta-regression analysis in North America showed that vaccination intentions were significantly higher between January and March 2021 (i.e., after vaccine release). This could be due to multiple factors, among them FDA approval of the first COVID-19 vaccine [130], as well as a number of social and political events that occurred in the meantime, including (but not limited to) the strongly pro-vax election campaign of current U.S. President Joe Biden [131] and the Canadian lockdown [132,133].

As for the methodological elements of the studies investigated, sample size and quality of the studies were not found to be relevant factors in estimating the prevalence of COVID-19 vaccination intentions. In contrast, the number of possible answers in the COVID-19 survey on vaccination intentions was found to influence the pooled estimates of vaccine acceptance. Specifically, investigating vaccine intentions through binary answers (i.e., offering the opportunity of answering “Yes or No” only) appears to be correlated with levels of vaccination acceptance higher than the acceptance estimates shown in studies using multi-point scales. The first methodological approach seems to be based on the evaluation of “vaccine behaviour” [134] instead of “vaccine intention” [134] and could underestimate levels of vaccine hesitancy. This phenomenon, by definition, is based on a continuum in the decision-making process [11] and is characterized by different levels of vaccination refusal or delay. Accordingly, it requires specific assessment tools that allow people to precisely state their own level of compliance with vaccinations: only being offered a “Yes” or “No” answer may in fact overestimate COVID-19 vaccination intentions (because “No” might only be a suitable response for individuals who refuse the vaccine outright) [135]. In fact, multiple studies have shown that administering response scales with a limited number of socially desirable categories increases the risk of receiving socially acceptable responses (e.g., in this case “Yes”). This phenomenon also occurs with the administration of scales/score that do not adequately differentiate responses at the extreme ends of the spectrum, and that do not allow individuals to understand the level at which to place their own behaviour [136,137,138]. Therefore, the use of validated instruments that consider the number of socially desirable categories would seem to be the best way to accurately identify the target population of hesitant individuals. Finally, a future implication for research could be studying the effect of the number of response categories, provided to assess vaccination hesitancy and intentions.

## 5. Conclusions

This study provides an insight into the potential resistance to vaccinations, investigated more than a year ago and in line with real-life data, offering valuable support to decision-makers to choose which public health strategies to implement. Indeed, despite some variation in the estimates, the meta-analysis showed that one in three people would refuse or delay COVID-19 vaccination, a proportion comparable to the current state of vaccination coverage. In addition, since levels of COVID-19 vaccine acceptance are currently below WHO thresholds, there is a pressing need to use validated tools to measure vaccine hesitancy to accurately identify hesitant individuals. Lastly, it is imperative that COVID-19 vaccination acceptance levels be monitored in lower-to-middle-income countries (e.g., Africa), where data are lacking and acceptance levels are below average.

## Figures and Tables

**Table 1:** Characteristics of the prevalence estimates included in the proportion meta-analysis of COVID-19 vaccination acceptance.

		*N (%)*
Geographical area		
	Africa	5 (3.9)
	Asia	31 (24.2)
	Europe	46 (35.9)
	North America	30 (23.4)
	Oceania	2 (1.6)
	South America	13 (10.2)
	Multiple countries	1 (0.8)
Target population		
	General Population	106 (82.8)
	Healthcare Workers	22 (17.2)
Time of investigation		
	March 2020–August 2020	65 (50.8)
	September 2020–December 2020	28 (21.9)
	January 2021–March 2021	28 (21.9)
	Not specified	7 (5.4)
Study quality		
	Poor/fair quality	88 (68.8)
	High quality	40 (31.2)
Type of answer options		
	Yes/No	40 (31.3)
	>2 choices	82 (64.0)
	Not Specified	6 (4.7)
Sample size		
	<1052 participants	64 (50.0)
	>1052 participants	64 (50.0)

**Table 2:** Pooled Prevalence Estimates (PEs) and their 95% Confidence Interval (CI) of COVID-19 vaccination acceptance by geographical area.

		N 5	Africa PE (95% CI) 0.36 (0.18–0.60)	N 31	Asia PE (95% CI) 0.66 (0.54–0.76)	N 46	Europe PE (95% CI) 0.71 (0.65–0.76)	N 30	North America PE (95% CI) 0.67 (0.59–0.74)	N 13	South America PE (95% CI) 0.81 (0.78–0.84)
Target population											
	General population	3	0.41 (0.10–0.82)	19	0.63 (0.50–0.74)	41	0.71 (0.64–0.77)	27	0.69 (0.61–0.76)	13	0.81 (0.78–0.84)
	Healthcare workers	2	0.33 (0.32–0.35)	12	0.70 (0.49–0.85)	5	0.65 (0.55–0.74)	3	0.53 (0.46–0.59)		-
Time of investigation											
	March 2020–August 2020	4	0.37 (0.14–0.68)	15	0.67 (0.51–0.80)	27	0.73 (0.66–0.79)	14	0.64 (0.56–0.72)	3	0.82 (0.77–0.86)
	September 2020–December 2020		-	10	0.74 (0.49–0.90)	13	0.69 (0.58–0.78)	4	0.55 (0.49–0.61)		-
	January 2021–March 2021	1	0.35 (0.33–0.37)	4	0.49 (0.31–0.67)	2	0.79 (0.75–0.83)	11	0.90 (0.87–0.92)	10	0.80 (0.80–0.80)
	Not specified		-	2	0.50 (0.48–0.52)	4	0.56 (0.43–0.68)	1	0.68 (0.63–0.74)		-
Study quality											
	Poor/fair quality	2	0.73 (0.71–0.75)	16	0.68 (0.51–0.78)	34	0.73 (0.66–0.79)	21	0.64 (0.52–0.75)	12	0.80 (0.80–0.80)
	High quality	3	0.25 (0.17–0.36)	15	0.63 (0.46–0.78)	12	0.68 (0.57–0.77)	9	0.69 (0.59–0.77)	1	0.85 (0.83–0.87)
Type of answer options											
	Yes/No	1	0.28 (0.24–0.31)	11	0.84 (0.66–0.94)	6	0.82 (0.74–0.88)	11	0.71 (0.35–0.92)	11	0.82 (0.79–0.86)
	>2 choices	2	0.73 (0.71–0.75)	20	0.55 (0.46–0.63)	39	0.67 (0.60–0.73)	16	0.69 (0.63–0.76)	2	0.79 (0.76–0.81)
	Not specified	2	0.24 (0.23–0.26)		-	1	0.86 (0.83–0.89)	3	0.49 (0.33–0.65)		-
Sample size											
	<1052 participants	3	0.51 (0.21–0.80)	19	0.64 (0.44–0.80)	29	0.73 (0.65–0.80)	10	0.62 (0.56–0.68)	3	0.82 (0.77–0.86)
	>1052 participants	2	0.24 (0.23–0.26)	12	0.67 (0.53–0.79)	17	0.69 (0.61–0.76)	20	0.68 (0.56–0.78)	10	0.80 (0.80–0.80)

**Table 3:** Multivariable meta-regression model predicting the pooled estimate of the prevalence of COVID-19 vaccination acceptance at the global level.

Variable Included in the Model		Meta−Regression Coefficient (95% CI)	SE	*p*−Value
Geographical areas				
	Europe (N = 46)	Ref.		
	Africa (N = 5)	−0.97 (−1.83 to −0.11)	0.43	0.027
	Asia (N = 31)	−0.02 (−0.42 to 0.39)	0.20	0.927
	North America (N = 30)	−0.01 (−0.43 to 0.41)	0.21	0.967
	Oceania (N = 2)	0.83 (−0.35 to 2.02)	0.60	0.166
	South America (N = 13)	−0.14 (−0.76 to 0.48)	0.31	0.657
	Multiple countries (N = 1)	−1.27 (−2.93 to 0.39)	0.84	0.131
Target population				
	General population (N = 106)	Ref.		
	Healthcare workers (N = 22)	−0.20 (−0.69 to 0.29)	0.25	0.425
Time of investigation				
	March 2020–August 2020 (N = 65)	Ref.		
	September 2020–December 2020 (N = 28)	−0.01 (−0.47 to 0.46)	0.23	0.977
	January 2021–March 2021 (N = 28)	−0.35 (−0.83 to 0.13)	0.24	0.155
	Not specified (N = 7)	−0.54 (−1.20 to 0.12)	0.33	0.110
Study quality				
	Poor/fair quality (N = 88)	Ref.		
	High quality (N = 40)	0.08 (−0.26 to 0.43)	0.17	0.631
Type of answer options				
	Yes/No (N = 40)	Ref.		
	>2 choices (N = 82)	−1.02 (−1.43 to −0.60)	0.21	< 0.001
	Not specified (N = 6)	−1.37 (−2.15 to −0.60)	0.39	0.001
Sample size				
	<1052 participants (N = 64)	Ref.		
	>1052 participants (N = 64)	−0.24 (−0.56 to 0.09)	0.16	0.149

CI: confidence interval. SE: standard error.

## Data Availability

Not applicable.

## Supplementary Materials

The following supporting information can be downloaded at: https://www.mdpi.com/article/10.3390/vaccines10091488/s1, Supplementary File S1: Table S1. Search Strategy; Supplementary Table S2. Multivariable meta-regression model predicting the pooled estimate of the Prevalence of COVID-19 vaccination intention by geographical area; Supplementary Figure S1. Forest plot of meta-analysis proportion (by country); Supplementary Figure S2. Forest plot of meta-analysis proportion (by geographical area); Supplementary File S2: PRISMA 2020 Checklist; PRISMA 2020 Abstract Checklist [139].

## References

[1] World Health Organization: Immunization Agenda 2030: A Global Strategy to Leave No One Behind. https://www.who.int/teams/immunization-vaccines-and-biologicals/strategies/ia2030.

[2] Dubé E., MacDonald N.E. (2022). COVID-19 vaccine hesitancy. Nat. Rev. Nephrol..

[3] Hussein I.E., Echams N., Echams S., Sayegh S.E., Badran R., Eraad M., Egerges-Geagea A., Eleone A., Ejurjus A. (2015). Vaccines Through Centuries: Major Cornerstones of Global Health. Front. Public Health.

[4] Our World in Data Coronavirus (COVID-19) Vaccinations. https://ourworldindata.org/covid-vaccinations.

[5] Achieving 70% COVID-19 Immunization Coverage by Mid-2022. https://www.who.int/news/item/23-12-2021-achieving-70-covid-19-immunization-coverage-by-mid-2022.

[6] Wouters O.J., Shadlen K.C., Salcher-Konrad M., Pollard A.J., Larson H.J., Teerawattananon Y., Jit M. (2021). Challenges in ensuring global access to COVID-19 vaccines: Production, affordability, allocation, and deployment. Lancet.

[7] Coronavirus Disease (COVID-19): Vaccine Access and Allocation. https://www.who.int/news-room/questions-and-answers/item/coronavirus-disease-(covid-19)-vaccine-access-and-allocation.

[8] Paul E., Steptoe A., Fancourt D. (2021). Attitudes towards vaccines and intention to vaccinate against COVID-19: Implications for public health communications. Lancet Reg. Health Eur..

[9] Razai M.S., Chaudhry U.A.R., Doerholt K., Bauld L., Majeed A. (2021). COVID-19 vaccination hesitancy. BMJ.

[10] Dror A.A., Eisenbach N., Taiber S., Morozov N.G., Mizrachi M., Zigron A., Srouji S., Sela E. (2020). Vaccine hesitancy: The next challenge in the fight against COVID-19. Eur. J. Epidemiol..

[11] MacDonald N.E., Eskola J., Liang X., Chaudhuri M., Dube E., Gellin B., Goldstein S., Larson H., Manzo M.L., Reingold A. (2015). Vaccine Hesitancy: Definition, Scope and Determinants. Vaccine.

[12] World Health Organization (WHO) (2019). Ten Threats to Global Health in 2019. https://www.who.int/news-room/spotlight/ten-threats-to-global-health-in-2019.

[13] Cascini F., Pantovic A., Al-Ajlouni Y., Failla G., Ricciardi W. (2021). Attitudes, acceptance and hesitancy among the general population worldwide to receive the COVID-19 vaccines and their contributing factors: A systematic review. eClinicalMedicine.

[14] Marcec R., Majta M., Likic R. (2020). Will vaccination refusal prolong the war on SARS-CoV-2?. Postgrad. Med. J..

[15] Dinga J.N., Sinda L.K., Titanji V.P.K. (2021). Assessment of Vaccine Hesitancy to a COVID-19 Vaccine in Cameroonian Adults and Its Global Implication. Vaccines.

[16] Lazarus J.V., Ratzan S.C., Palayew A., Gostin L.O., Larson H.J., Rabin K., Kimball S., El-Mohandes A. (2021). A global survey of potential acceptance of a COVID-19 vaccine. Nat. Med..

[17] Al-Qerem W.A., Jarab A.S. (2021). COVID-19 Vaccination Acceptance and Its Associated Factors Among a Middle Eastern Population. Front. Public Health.

[18] Dubé E., MacDonald N.E. (2020). How can a global pandemic affect vaccine hesitancy?. Expert Rev. Vaccines.

[19] Lin C., Tu P., Beitsch L.M. (2021). Confidence and Receptivity for COVID-19 Vaccines: A Rapid Systematic Review. Vaccines.

[20] Norhayati M.N., Che Yusof R., Azman Y.M. (2022). Systematic Review and Meta-Analysis of COVID-19 Vaccination Acceptance. Front. Med..

[21] Gholami M., Fawad I., Shadan S., Rowaiee R., Ghanem H., Khamis A.H., Ho S.B. (2021). COVID-19 and healthcare workers: A systematic review and meta-analysis. Int. J. Infect. Dis..

[22] Luo C., Yang Y., Liu Y., Zheng D., Shao L., Jin J., He Q. (2021). Intention to COVID-19 vaccination and associated factors among health care workers: A systematic review and meta-analysis of cross-sectional studies. Am. J. Infect. Control.

[23] Al-Amer R., Maneze D., Everett B., Montayre J., Villarosa A.R., Dwekat E., Salamonson Y. (2021). COVID-19 vaccination intention in the first year of the pandemic: A systematic review. J. Clin. Nurs..

[24] Wang Q., Yang L., Jin H., Lin L. (2021). Vaccination against COVID-19: A systematic review and meta-analysis of acceptability and its predictors. Prev. Med..

[25] Chen F., He Y., Shi Y. (2022). Parents’ and Guardians’ Willingness to Vaccinate Their Children against COVID-19: A Systematic Review and Meta-Analysis. Vaccines.

[26] Shamshirsaz A.A., Hessami K., Morain S., Afshar Y., Nassr A.A., Arian S.E., Asl N.M., Aagaard K. (2021). Intention to Receive COVID-19 Vaccine during Pregnancy: A Systematic Review and Meta-analysis. Am. J. Perinatol..

[27] Moher D., Liberati A., Tetzlaff J., Altman D.G., Altman D., Antes G., Atkins D., Barbour V., Barrowman N., Berlin J.A. (2009). Preferred Reporting Items for Systematic Reviews and Meta-Analyses: The PRISMA Statement. PLoS Med..

[28] Dorman C., Perera A., Condon C., Chau C., Qian J., Kalk K., DiazDeleon D. (2021). Factors Associated with Willingness to be Vaccinated Against COVID-19 in a Large Convenience Sample. J. Community Health.

[29] Hillen M.A., Medendorp N.M., Daams J.G., Smets E.M. (2017). Patient-Driven Second Opinions in Oncology: A Systematic Review. Oncologist.

[30] Lin L., Chu H. (2020). Meta-analysis of Proportions Using Generalized Linear Mixed Models. Epidemiology.

[31] Higgins J.P.T., Thompson S.G., Deeks J.J., Altman D.G. (2003). Measuring inconsistency in meta-analyses. BMJ.

[32] Akarsu B., Canbay Özdemir D., Ayhan Baser D., Aksoy H., Fidancı İ., Cankurtaran M. (2021). While studies on COVID-19 vaccine is ongoing, the public’s thoughts and attitudes to the future COVID-19 vaccine. Int. J. Clin. Pract..

[33] Alabdulla M., Reagu S.M., Al-Khal A., Elzain M., Jones R.M. (2021). COVID-19 vaccine hesitancy and attitudes in Qatar: A national cross-sectional survey of a migrant-majority population. Influenza Other Respir. Viruses.

[34] Alfageeh E.I., Alshareef N., Angawi K., Alhazmi F., Chirwa G.C. (2021). Acceptability of a COVID-19 Vaccine among the Saudi Population. Vaccines.

[35] Al-Mohaithef M., Padhi B.K. (2020). Determinants of COVID-19 Vaccine Acceptance in Saudi Arabia: A Web-Based National Survey. J. Multidiscip. Health.

[36] Barello S., Nania T., Dellafiore F., Graffigna G., Caruso R. (2020). ‘Vaccine hesitancy’ among university students in Italy during the COVID-19 pandemic. Eur. J. Epidemiol..

[37] Bell S., Clarke R., Mounier-Jack S., Walker J.L., Paterson P. (2020). Parents’ and guardians’ views on the acceptability of a future COVID-19 vaccine: A multi-methods study in England. Vaccine.

[38] Biasio L.R., Bonaccorsi G., Lorini C., Pecorelli S., Roberto L., Bonaccorsi G., Lorini C., Pecorelli S., Biasio L.R., Bonac-corsi G. (2020). Assessing COVID-19 vaccine literacy: A preliminary online survey. Hum. Vaccines Immunother..

[39] Biasio L., Bonaccorsi G., Lorini C., Mazzini D., Pecorelli S. (2021). Italian Adults’ Likelihood of Getting COVID-19 Vaccine: A Second Online Survey. Vaccines.

[40] Chew N.W., Cheong C., Kong G., Phua K., Ngiam J.N., Tan B.Y., Wang B., Hao F., Tan W., Han X. (2021). An Asia-Pacific study on healthcare workers’ perceptions of, and willingness to receive, the COVID-19 vaccination. Int. J. Infect. Dis..

[41] Cordina M., Lauri M.A., Lauri J. (2021). Attitudes towards COVID-19 vaccination, vaccine hesitancy and intention to take the vaccine. Pharm. Pract..

[42] Detoc M., Bruel S., Frappe P., Tardy B., Botelho-Nevers E., Gagneux-Brunon A. (2020). Intention to participate in a COVID-19 vaccine clinical trial and to get vaccinated against COVID-19 in France during the pandemic. Vaccine.

[43] Di Gennaro F., Murri R., Segala F.V., Cerruti L., Abdulle A., Saracino A., Bavaro D.F., Fantoni M. (2021). Attitudes towards Anti-SARS-CoV2 Vaccination among Healthcare Workers: Results from a National Survey in Italy. Viruses.

[44] Dodd R.H., Cvejic E., Bonner C., Pickles K., McCaffery K.J., Ayre J., Batcup C., Copp T., Cornell S., Dakin T. (2020). Willingness to vaccinate against COVID-19 in Australia. Lancet Infect. Dis..

[45] Eguia H., Vinciarelli F., Bosque-Prous M., Kristensen T., Saigí-Rubió F. (2021). Spain’s Hesitation at the Gates of a COVID-19 Vaccine. Vaccines.

[46] Ehde D.M., Roberts M.K., Herring T.E., Alschuler K.N. (2021). Willingness to obtain COVID-19 vaccination in adults with multiple sclerosis in the United States. Mult. Scler. Relat. Disord..

[47] Fisher K.A., Bloomstone S.J., Walder J., Crawford S., Fouayzi H., Mazor K.M. (2020). Attitudes toward a potential SARS-CoV-2 vaccine: A survey of U.S. adults. Ann. Intern. Med..

[48] Freeman D., Waite F., Rosebrock L., Petit A., Causier C., East A., Jenner L., Teale A.-L., Carr L., Mulhall S. (2020). Coronavirus conspiracy beliefs, mistrust, and compliance with government guidelines in England. Psychol. Med..

[49] Freeman D., Loe B.S., Chadwick A., Vaccari C., Waite F., Rosebrock L., Jenner L., Petit A., Lewandowsky S., Vanderslott S. (2020). COVID-19 vaccine hesitancy in the UK: The Oxford coronavirus explanations, attitudes, and narratives survey (Oceans) II. Psychol. Med..

[50] Gagneux-Brunon A., Detoc M., Bruel S., Tardy B., Rozaire O., Frappe P., Botelho-Nevers E. (2021). Intention to get vaccinations against COVID-19 in French healthcare workers during the first pandemic wave: A cross-sectional survey. J. Hosp. Infect..

[51] Gerussi V., Peghin M., Palese A., Bressan V., Visintini E., Bontempo G., Graziano E., de Martino M., Isola M., Tascini C. (2021). Vaccine Hesitancy among Italian Patients Recovered from COVID-19 Infection towards Influenza and Sars-CoV-2 Vaccination. Vaccines.

[52] Graffigna G., Palamenghi L., Barello S., Stefania B. (2020). “Cultivating” acceptance of a COVID-19 vaccination program: Lessons from Italy. Vaccine.

[53] Grech V., Gauci C., Agius S. (2020). Withdrawn: Vaccine hesitancy among Maltese Healthcare workers toward influenza and novel COVID-19 vaccination. Early Hum. Dev..

[54] Head K.J., Kasting M.L., Sturm L.A., Hartsock J.A., Zimet G.D. (2020). A National Survey Assessing SARS-CoV-2 Vaccination Intentions: Implications for Future Public Health Communication Efforts. Sci. Commun..

[55] Kabamba Nzaji M., Kabamba Ngombe L., Ngoie Mwamba G., Banza Ndala D.B., Mbidi Miema J., Lungoyo C.L., Mwimba B.L., Bene A.C.M., Musenga E.M. (2020). Acceptability of Vaccination Against COVID-19 Among Healthcare Workers in the Democratic Republic of the Congo. Pragmatic Obs. Res..

[56] Kaplan A.K., Sahin M.K., Parildar H., Guvenc I.A. (2021). The willingness to accept the COVID-19 vaccine and affecting factors among healthcare professionals: A cross-sectional study in Turkey. Int. J. Clin. Pract..

[57] Khubchandani J., Sharma S., Price J.H., Wiblishauser M.J., Sharma M., Webb F.J. (2021). COVID-19 Vaccination Hesitancy in the United States: A Rapid National Assessment. J. Community Health.

[58] Kociolek L.K., Elhadary J., Jhaveri R., Patel A.B., Stahulak B., Cartland J. (2021). Coronavirus disease 2019 vaccine hesitancy among children’s hospital staff: A single-center survey. Infect. Control Hosp. Epidemiol..

[59] Kourlaba G., Kourkouni E., Maistreli S., Tsopela C.-G., Molocha N.-M., Triantafyllou C., Koniordou M., Kopsidas I., Chorianopoulou E., Maroudi-Manta S. (2021). Willingness of Greek general population to get a COVID-19 vaccine. Glob. Health Res. Policy.

[60] Kuppalli K., Brett-Major D.M., Smith T.C. (2021). COVID-19 Vaccine Acceptance: We Need to Start Now. Open Forum Infect. Dis..

[61] Kwok K.O., Li K.-K., Wei W.I., Tang A., Wong S.Y.S., Lee S.S. (2021). Influenza vaccine uptake, COVID-19 vaccination intention and vaccine hesitancy among nurses: A survey. Int. J. Nurs. Stud..

[62] Latkin C.A., Dayton L., Yi G., Colon B., Kong X. (2021). Mask usage, social distancing, racial, and gender correlates of COVID-19 vaccine intentions among adults in the US. PLoS ONE.

[63] Lin Y., Hu Z., Zhao Q., Alias H., Danaee M., Wong L.P. (2020). Understanding COVID-19 vaccine demand and hesitancy: A nationwide online survey in China. PLoS Negl. Trop. Dis..

[64] Meyer M.N., Gjorgjieva T., Rosica D. (2021). Trends in Health Care Worker Intentions to Receive a COVID-19 Vaccine and Reasons for Hesitancy. JAMA Netw. Open.

[65] Murphy J., Vallières F., Bentall R.P., Shevlin M., McBride O., Hartman T.K., McKay R., Bennett K., Mason L., Gibson-Miller J. (2021). Psychological characteristics associated with COVID-19 vaccine hesitancy and resistance in Ireland and the United Kingdom. Nat. Commun..

[66] Nguyen L.H., Joshi A.D., Drew D.A., Merino J., Ma W., Lo C.-H., Kwon S., Wang K., Graham M.S., Polidori L. (2022). Self-reported COVID-19 vaccine hesitancy and uptake among participants from different racial and ethnic groups in the United States and United Kingdom. Nat. Commun..

[67] Qattan A.M.N., Alshareef N., Alsharqi O., Al Rahahleh N., Chirwa G.C., Al-Hanawi M.K. (2021). Acceptability of a COVID-19 Vaccine Among Healthcare Workers in the Kingdom of Saudi Arabia. Front. Med..

[68] Palamenghi L., Barello S., Boccia S., Graffigna G. (2020). Mistrust in biomedical research and vaccine hesitancy: The forefront challenge in the battle against COVID-19 in Italy. Eur. J. Epidemiol..

[69] Petravić L., Arh R., Gabrovec T., Jazbec L., Rupčić N., Starešinič N., Zorman L., Pretnar A., Srakar A., Zwitter M. (2021). Factors Affecting Attitudes towards COVID-19 Vaccination: An Online Survey in Slovenia. Vaccines.

[70] Pogue K., Jensen J.L., Stancil C.K., Ferguson D.G., Hughes S.J., Mello E.J., Burgess R., Berges B.K., Quaye A., Poole B.D. (2020). Influences on Attitudes Regarding Potential COVID-19 Vaccination in the United States. Vaccines.

[71] Reiter P.L., Pennell M.L., Katz M.L. (2020). Acceptability of a COVID-19 vaccine among adults in the United States: How many people would get vaccinated?. Vaccine.

[72] Rhodes A., Hoq M., Measey M.-A., Danchin M. (2020). Intention to vaccinate against COVID-19 in Australia. Lancet Infect. Dis..

[73] Ruiz J.B., Bell R.A. (2021). Predictors of intention to vaccinate against COVID-19: Results of a nationwide survey. Vaccine.

[74] Saied S.M., Saied E.M., Kabbash I.A., Abdo S.A.E. (2021). Vaccine hesitancy: Beliefs and barriers associated with COVID-19 vaccination among Egyptian medical students. J. Med. Virol..

[75] Salali G.D., Uysal M.S. (2020). COVID-19 vaccine hesitancy is associated with beliefs on the origin of the novel coronavirus in the UK and Turkey. Psychol. Med..

[76] Sarasty O., Carpio C.E., Hudson D., Guerrero-Ochoa P.A., Borja I. (2020). The demand for a COVID-19 vaccine in Ecuador. Vaccine.

[77] Schwarzinger M., Watson V., Arwidson P., Alla F., Luchini S. (2021). COVID-19 vaccine hesitancy in a representative working-age population in France: A survey experiment based on vaccine characteristics. Lancet Public Health.

[78] Serrazina F., Pinho A.S., Cabral G., Salavisa M., Correia A.S. (2021). Willingness to be vaccinated against COVID-19: An exploratory online survey in a Portuguese cohort of multiple sclerosis patients. Mult. Scler. Relat. Disord..

[79] Sherman S.M., Smith L.E., Sim J., Amlôt R., Cutts M., Dasch H., Rubin G.J., Sevdalis N. (2020). COVID-19 vaccination intention in the UK: Results from the COVID-19 vaccination acceptability study (CoVAccS), a nationally representative cross-sectional survey. Hum. Vaccines Immunother..

[80] Soares P., Moniz M., Gama A., Laires P.A., Pedro A.R., Dias S., Leite A., Nunes C. (2021). Factors Associated with COVID-19 Vaccine Hesitancy. Vaccines.

[81] Taylor S., Landry C.A., Paluszek M.M., Groenewoud R., Rachor G.S., Asmundson G.J.G. (2020). A Proactive Approach for Managing COVID-19: The Importance of Understanding the Motivational Roots of Vaccination Hesitancy for SARS-CoV2. Front. Psychol..

[82] Unroe K.T., Evans R., Weaver L., Rusyniak D., Blackburn J. (2020). Willingness of Long-Term Care Staff to Receive a COVID -19 Vaccine: A Single State Survey. J. Am. Geriatr. Soc..

[83] Urrunaga-Pastor D., Bendezu-Quispe G., Herrera-Añazco P., Uyen-Cateriano A., Toro-Huamanchumo C.J., Rodriguez-Morales A.J., Hernandez A.V., Benites-Zapata V.A. (2021). Cross-sectional analysis of COVID-19 vaccine intention, perceptions and hesitancy across Latin America and the Caribbean. Travel Med. Infect. Dis..

[84] Vallée A., Fourn E., Majerholc C., Touche P., Zucman D. (2021). COVID-19 Vaccine Hesitancy among French People Living with HIV. Vaccines.

[85] Wang K., Wong E.L.Y., Ho K.F., Cheung A.W.L., Chan E.Y.Y., Yeoh E.K., Wong S.Y.S. (2020). Intention of nurses to accept coronavirus disease 2019 vaccination and change of intention to accept seasonal influenza vaccination during the coronavirus disease 2019 pandemic: A cross-sectional survey. Vaccine.

[86] Wang J., Jing R., Lai X., Zhang H., Lyu Y., Knoll M.D., Fang H. (2020). Acceptance of COVID-19 Vaccination during the COVID-19 Pandemic in China. Vaccines.

[87] Ward J.K., Alleaume C., Peretti-Watel P., Seror V., Cortaredona S., Launay O., Raude J., Verger P., Beck F., Legleye S. (2020). The French public’s attitudes to a future COVID-19 vaccine: The politicization of a public health issue. Soc. Sci. Med..

[88] Williams L., Gallant A.J., Rasmussen S., Nicholls L.A.B., Cogan N., Deakin K., Young D., Flowers P. (2020). Towards intervention development to increase the uptake of COVID-19 vaccination among those at high risk: Outlining evidence-based and theoretically informed future intervention content. Br. J. Health Psychol..

[89] Yurttas B., Poyraz B.C., Sut N., Ozdede A., Oztas M., Uğurlu S., Tabak F., Hamuryudan V., Seyahi E. (2021). Willingness to get the COVID-19 vaccine among patients with rheumatic diseases, healthcare workers and general population in Turkey: A web-based survey. Rheumatol. Int..

[90] Wong L.P., Alias H., Wong P.-F., Lee H.Y., Abubakar S. (2020). The use of the health belief model to assess predictors of intent to receive the COVID-19 vaccine and willingness to pay. Hum. Vaccines Immunother..

[91] Ledda C., Costantino C., Cuccia M., Maltezou H.C., Rapisarda V. (2021). Attitudes of Healthcare Personnel towards Vaccinations before and during the COVID-19 Pandemic. Int. J. Environ. Res. Public Health.

[92] Machida M., Nakamura I., Kojima T., Saito R., Nakaya T., Hanibuchi T., Takamiya T., Odagiri Y., Fukushima N., Kikuchi H. (2021). Acceptance of a COVID-19 Vaccine in Japan during the COVID-19 Pandemic. Vaccines.

[93] Malik A.A., McFadden S.M., Elharake J., Omer S.B. (2020). Determinants of COVID-19 vaccine acceptance in the US. eClinicalMedicine.

[94] Muqattash R., Niankara I., Traoret R.I. (2020). Survey data for COVID-19 vaccine preference analysis in the United Arab Emirates. Data Brief.

[95] Neumann-Böhme S., Varghese N.E., Sabat I., Barros P.P., Brouwer W., van Exel J., Schreyögg J., Stargardt T. (2020). Once we have it, will we use it? A European survey on willingness to be vaccinated against COVID-19. Eur. J. Health Econ..

[96] Graffigna G., Palamenghi L., Boccia S., Barello S. (2020). Relationship between Citizens’ Health Engagement and Intention to Take the COVID-19 Vaccine in Italy: A Mediation Analysis. Vaccines.

[97] COVID-19 Vaccination—Africa CDC. https://africacdc.org/covid-19-vaccination/.

[98] Tagoe E.T., Sheikh N., Morton A., Nonvignon J., Sarker A.R., Williams L., Megiddo I. (2021). COVID-19 Vaccination in Lower-Middle Income Countries: National Stakeholder Views on Challenges, Barriers, and Potential Solutions. Front. Public Health.

[99] OECD Access to COVID-19 Vaccines: Global Approaches in a Global Crisis—OECD. https://read.oecd-ilibrary.org/view/?ref=1069_1069384-ewmqrw9sx2&title=Access-to-COVID-19-vaccines-Global-approaches-in-a-global-crisis.

[100] WHO COVAX. https://www.who.int/initiatives/act-accelerator/covax.

[101] Simas C., Larson H.J. (2021). Overcoming vaccine hesitancy in low-income and middle-income regions. Nat. Rev. Dis. Prim..

[102] Cadeddu C., Regazzi L., Bonaccorsi G., Rosano A., Unim B., Griebler R., Link T., de Castro P., D’Elia R., Mastrilli V. (2022). The Determinants of Vaccine Literacy in the Italian Population: Results from the Health Literacy Survey 2019. Int. J. Environ. Res. Public Health.

[103] Mutombo P.N., Fallah M.P., Munodawafa D., Kabel A., Houeto D., Goronga T., Mweemba O., Balance G., Onya H., Kamba R.S. (2021). COVID-19 vaccine hesitancy in Africa: A call to action. Lancet Glob. Health.

[104] Baccolini V., Rosso A., di Paolo C., Isonne C., Salerno C., Migliara G., Prencipe G.P., Massimi A., Marzuillo C., de Vito C. (2021). What is the Prevalence of Low Health Literacy in European Union Member States? A Systematic Review and Meta-analysis. J. Gen. Intern. Med..

[105] Nature Africa COVID-19 Vaccine Uptake in Africa. https://www.nature.com/articles/d44148-022-00003-0.

[106] Patwary M.M., Alam A., Bardhan M., Disha A.S., Haque Z., Billah S.M., Kabir P., Browning M.H.E.M., Rahman M., Parsa A.D. (2022). COVID-19 Vaccine Acceptance among Low- and Lower-Middle-Income Countries: A Rapid Systematic Review and Meta-Analysis. Vaccines.

[107] Larson H.J., Gakidou E., Murray C.J.L. (2022). The Vaccine-Hesitant Moment. N. Engl. J. Med..

[108] Li M., Luo Y., Watson R., Zheng Y., Ren J., Tang J., Chen Y. (2021). Healthcare workers’ (HCWs) attitudes and related factors towards COVID-19 vaccination: A rapid systematic review. Postgrad. Med. J..

[109] Ackah M., Ameyaw L., Salifu M.G., Asubonteng D.P.A., Yeboah C.O., Annor E.N., Ankapong E.A.K., Boakye H. (2022). COVID-19 vaccine acceptance among health care workers in Africa: A systematic review and meta-analysis. PLoS ONE.

[110] Petek D., Kamnik-Jug K. (2018). Motivators and barriers to vaccination of health professionals against seasonal influenza in primary healthcare. BMC Health Serv. Res..

[111] Genovese C., Picerno I., Trimarchi G., Cannavò G., Egitto G., Cosenza B., Merlina V., Icardi G., Panatto D., Amicizia D. (2019). Vaccination coverage in healthcare workers: A multicenter cross-sectional study in Italy. J. Prev. Med. Hyg..

[112] Wilson R., Scronias D., Zaytseva A., Ferry M.-A., Chamboredon P., Dubé E., Verger P. (2019). Seasonal influenza self-vaccination behaviours and attitudes among nurses in Southeastern France. Hum. Vaccines Immunother..

[113] Paoli S., Lorini C., Puggelli F., Sala A., Grazzini M., Paolini D., Bonanni P., Bonaccorsi G. (2019). Assessing Vaccine Hesitancy among Healthcare Workers: A Cross-Sectional Study at an Italian Paediatric Hospital and the Development of a Healthcare Worker’s Vaccination Compliance Index. Vaccines.

[114] Pitini E., Baccolini V., Rosso A., Massimi A., de Vito C., Marzuillo C., Villari P. (2021). How Public Health Professionals View Mandatory Vaccination in Italy—A Cross-Sectional Survey. Vaccines.

[115] Sindoni A., Baccolini V., Adamo G., Massimi A., Migliara G., de Vito C., Marzuillo C., Villari P. (2021). Effect of the mandatory vaccination law on measles and rubella incidence and vaccination coverage in Italy (2013–2019). Hum. Vaccines Immunother..

[116] Dzieciolowska S., Hamel D., Gadio S., Dionne M., Gagnon D., Robitaille L., Cook E., Caron I., Talib A., Parkes L. (2021). COVID-19 vaccine acceptance, hesitancy, and refusal among Canadian healthcare workers: A multicenter survey. Am. J. Infect. Control.

[117] Manby L., Dowrick A., Karia A., Maio L., Buck C., Singleton G., Lewis-Jackson S., Uddin I., Vanderslott S., Martin S. (2022). Healthcare workers’ perceptions and attitudes towards the UK’s COVID-19 vaccination programme: A rapid qualitative appraisal. BMJ Open.

[118] Rutten L.J.F., Zhu X., Leppin A.L., Ridgeway J.L., Swift M.D., Griffin J.M., Sauver J.L.S., Virk A., Jacobson R.M. (2021). Evidence-Based Strategies for Clinical Organizations to Address COVID-19 Vaccine Hesitancy. Mayo Clin. Proc..

[119] Fakonti G., Kyprianidou M., Iordanou S., Toumbis G., Giannakou K. (2022). General vaccination knowledge influences nurses’ and midwives’ COVID-19 vaccination intention in Cyprus: A nationwide cross-sectional study. Hum. Vaccines Immunother..

[120] Huang Y., Su X., Xiao W., Wang H., Si M., Wang W., Gu X., Ma L., Li L., Zhang S. (2022). COVID-19 vaccine hesitancy among different population groups in China: A national multicenter online survey. BMC Infect. Dis..

[121] Liu H., Zhou Z., Tao X., Huang L., Zhu E., Yu L., Du S., Zhang M. (2022). Willingness and Influencing Factors to Receive COVID-19 Vaccination Among Chinese Medical Students. Front. Public Health.

[122] Lazarus J.V., Wyka K., White T.M., Picchio C.A., Rabin K., Ratzan S.C., Leigh J.P., Hu J., El-Mohandes A. (2022). Revisiting COVID-19 vaccine hesitancy around the world using data from 23 countries in 2021. Nat. Commun..

[123] Baccolini V., Renzi E., Isonne C., Migliara G., Massimi A., de Vito C., Marzuillo C., Villari P. (2021). COVID-19 Vaccine Hesitancy among Italian University Students: A Cross-Sectional Survey during the First Months of the Vaccination Campaign. Vaccines.

[124] Schuster M., Eskola J., Duclos P., Liang X., Chaudhuri M., Dube E., Gellin B., Goldstein S., Larson H., MacDonald N. (2015). Review of vaccine hesitancy: Rationale, remit and methods. Vaccine.

[125] Siddiqui M., Salmon D.A., Omer S.B. (2013). Epidemiology of vaccine hesitancy in the United States. Hum. Vaccines Immunother..

[126] Karim M.A., Reagu S.M., Ouanes S., Khan A.W., Smidi W.S., Al-Baz N., Alabdulla M. (2022). Prevalence and correlates of COVID-19 vaccine hesitancy among the elderly in Qatar: A cross-sectional study. Medicine.

[127] Tsang S.J. (2022). Predicting COVID-19 vaccine hesitancy in Hong Kong: Vaccine knowledge, risks from coronavirus, and risks and benefits of vaccination. Vaccine X.

[128] Roy D.N., Biswas M., Islam E., Azam S. (2022). Potential factors influencing COVID-19 vaccine acceptance and hesitancy: A systematic review. PLoS ONE.

[129] Qiao S., Li Z., Liang C., Li X., Rudisill C. (2022). Three dimensions of COVID-19 risk perceptions and their socioeconomic correlates in the United States: A social media analysis. Risk Anal..

[130] FDA Approves First COVID-19 Vaccine|FDA. https://www.fda.gov/news-events/press-announcements/fda-approves-first-covid-19-vaccine.

[131] Biden, Trump Battle over Prospect of Coronavirus Vaccine Delivered before Election Day. https://www.nbcnews.com/politics/2020-election/biden-trump-battle-over-prospect-coronavirus-vaccine-delivered-election-day-n1239479.

[132] Toronto Lockdown—One of the World’s Longest?—BBC News. https://www.bbc.com/news/world-us-canada-57079577.

[133] Ontario Announces Hard Lockdown after Covid Cases Surge|Canada|The Guardian. https://www.theguardian.com/world/2020/dec/21/ontario-canada-announces-hard-lockdown-after-covid-cases-surge.

[134] Betsch C., Böhm R., Chapman G.B., Fiske S.T. (2015). Using Behavioral Insights to Increase Vaccination Policy Effectiveness. Policy Insights Behav. Brain Sci..

[135] Larson H.J., Jarrett C., Schulz W.S., Chaudhuri M., Zhou Y., Dubé E., Schuster M., MacDonald N.E., Wilson R., The SAGE Working Group on Vaccine Hesitancy (2015). Measuring vaccine hesitancy: The development of a survey tool. Vaccine.

[136] Moors G. (2007). Exploring the effect of a middle response category on response style in attitude measurement. Qual. Quant..

[137] Rockwood T.H., Sangster R.L., Dillman D.A. (1997). The Effect of Response Categories on Questionnaire Answers: Context an Mode Effects. Sociol. Methods Res..

[138] Schwarz N., Hippler H.J., Deutsch B., Strack F. (1985). Response Scales: Effects of Category Range on Reported Behavior and Comparative Judgments. Public Opin. Q..

[139] Page M.J., McKenzie J.E., Bossuyt P.M., Boutron I., Hoffmann T.C., Mulrow C.D., Shamseer L., Tetzlaff J.M., Akl E.A., Brennan S.E. (2021). The PRISMA 2020 statement: An updated guideline for reporting systematic reviews. BMJ.

