# Supplementary material for: Mapping the Prevalence of COVID-19 Vaccine Acceptance at the Global and Regional Level: A Systematic Review and Meta-Analysis
Source: Vaccines (Basel). 2022 Sep 7;10(9):1488. doi: 10.3390/vaccines10091488 (PMC9506365; doi:10.3390/vaccines10091488)
Supplement: Supplementary file 1 [file vaccines-10-01488-s001.zip › Supplementary File S1.pdf]

Supplementary Table S1. Search Strategy

Database	Search Strategy	N
PubMed	(vaccin* [Title/Abstract]) AND (hesitancy[Title/Abstract] OR reluctance[Title/Abstract] OR resistance[Title/Abstract] OR refusal[Title/Abstract] OR acceptance[Title/Abstract] OR willingness[Title/Abstract] OR behavi*[Title/Abstract] OR uptake[Title/Abstract] OR rate*[Title/Abstract] OR recei*[Title/Abstract] OR aware*[Title/Abstract] OR coverage[Title/Abstract] OR barriers[Title/Abstract] OR factor*[Title/Abstract] OR opinion[Title/Abstract] OR determinant[Title/Abstract] OR respons*[Title/Abstract] OR predictor[Title/Abstract] OR intention[Title/Abstract] OR skeptic*[Title/Abstract] OR perception*[Title/Abstract] OR intention*[Title/Abstract] OR attitude*[Title/Abstract] OR decision[Title/Abstract] OR belief*[Title/Abstract] OR knowledge[Title/Abstract]) AND (COVID-19[Title/Abstract] OR SARS-CoV-2[Title/Abstract] OR "coronavirus disease 2019"[Title/Abstract] OR "severe acute respiratory syndrome coronavirus 2"[Title/Abstract] OR 2019-ncov[Title/Abstract])	2051
PsycInfo	AB (vaccin*) AND (hesitancy OR reluctance OR resistance OR refusal OR acceptance OR willingness OR behavi* OR uptake OR rate* OR recei* OR aware* OR coverage OR barriers OR factor* OR opinion OR determinant OR respons* OR predictor OR intention OR skeptic* OR perception* OR intention*OR attitude* OR decision OR belief*OR knowledge) AND (COVID-19 OR SARS-CoV-2 OR "coronavirus disease 2019”OR "severe acute respiratory syndrome coronavirus 2" OR 2019-ncov)	36
Scopus	TITLE-ABS (vaccin*) AND TITLE-ABS (hesitancy OR reluctance OR resistance OR refusal OR acceptance OR willingness OR behavi* OR uptake OR rate* OR recei* OR aware* OR coverage OR barriers OR factor* OR opinion OR determinant OR respons* OR predictor OR intention OR skeptic* OR perception* OR intention* OR attitude* OR decision OR belief* OR knowledge) AND TITLE-ABS (covid-19 OR sars-cov-2 OR "coronavirus disease 2019" OR "severe acute respiratory syndrome coronavirus 2" OR 2019-ncov)	1857
Web Of Science	TOPIC: ((vaccin*) AND (hesitancy OR reluctance OR resistance OR refusal OR acceptance OR willingness OR behavi* OR uptake OR rate* OR recei* OR aware* OR coverage OR barriers OR factor* OR opinion OR determinant OR respons* OR predictor OR intention OR skeptic* OR perception* OR intention*OR attitude* OR decision OR belief*OR knowledge) AND (COVID-19 OR SARS-CoV-2 OR "coronavirus disease 2019"OR "severe acute respiratory syndrome coronavirus 2" OR 2019-ncov))	1503

Supplementary Table S2. Multivariable meta-regression model predicting the pooled estimate of the Prevalence of COVID-19 vaccination intention by geographical area

	Asia		Europe		North America	
	Meta-regression coefficient (95% CI)	P-value	Meta-regression coefficient (95% CI)	P-value	Meta-regression coefficient (95% CI)	P-value
Target population						
General population	Ref.		Ref.		Ref.	
Healthcare workers	0.68 (- 0 .96 to 1.10)	0.893	-0.42 (- 1.14 to 0.30)	0.249	-1.06 (- 2.48 to 0.36)	0.135
Time of investigation						
March 2020 – August 2020	Ref.		Ref.		Ref.	
September 2020 – December 2020	- 0.88 (- 2.55 to 0.78)	0.283	- 0.14 (- 0.64 to 0.34)	0.543	0.81 (- 0.59 to 2.21)	0.243
January 2021 – March 2021	- 0.95 (- 2.12 to 0.24)	0.112	0.33 (- 0.69 to 1.35)	0.518	1.36 (0.47 to 2.25)	0.005
Not specified	- 0.87 (- 2.66 to 0.92)	0.324	- 0.47 (- 1.22 to 0.27)	0.207	0.10 (- 1.50 to 1.24)	0.864
Study quality						
Poor/fair quality	Ref.		Ref.		Ref.	
High quality	0.49 (- 0.62 to 1.60)	0.371	0.01 (- 0.54 to 0.52)	0.969	0.20 (- 0.75 to 0.35)	0.452
Type of answer options						
Yes/No	Ref.		Ref.		Ref.	
> 2 choices	- 2.11 (- 3.44 to - 0.79)	0.003	- 0.82 (- 1.44 to - 0.20)	0.010	1.16 (0.26 to 2.06)	0.014
Not specified	--		0.17 (- 1.27 to 1.61)	0.812	- 0.17 (- 1.22 to 0.89)	0.745
Sample size						
< 1052 participants	Ref.		Ref.		Ref.	
> 1052 participants	- 0.63 (- 1.53 to 0.26)	0.157	- 0.00 (- 0.47 to 0.47)	0.993	0.51 (- 0.00 to 1.02)	0.050

Supplementary Figure S1. Forest plot of meta-analysis proportion (by country)

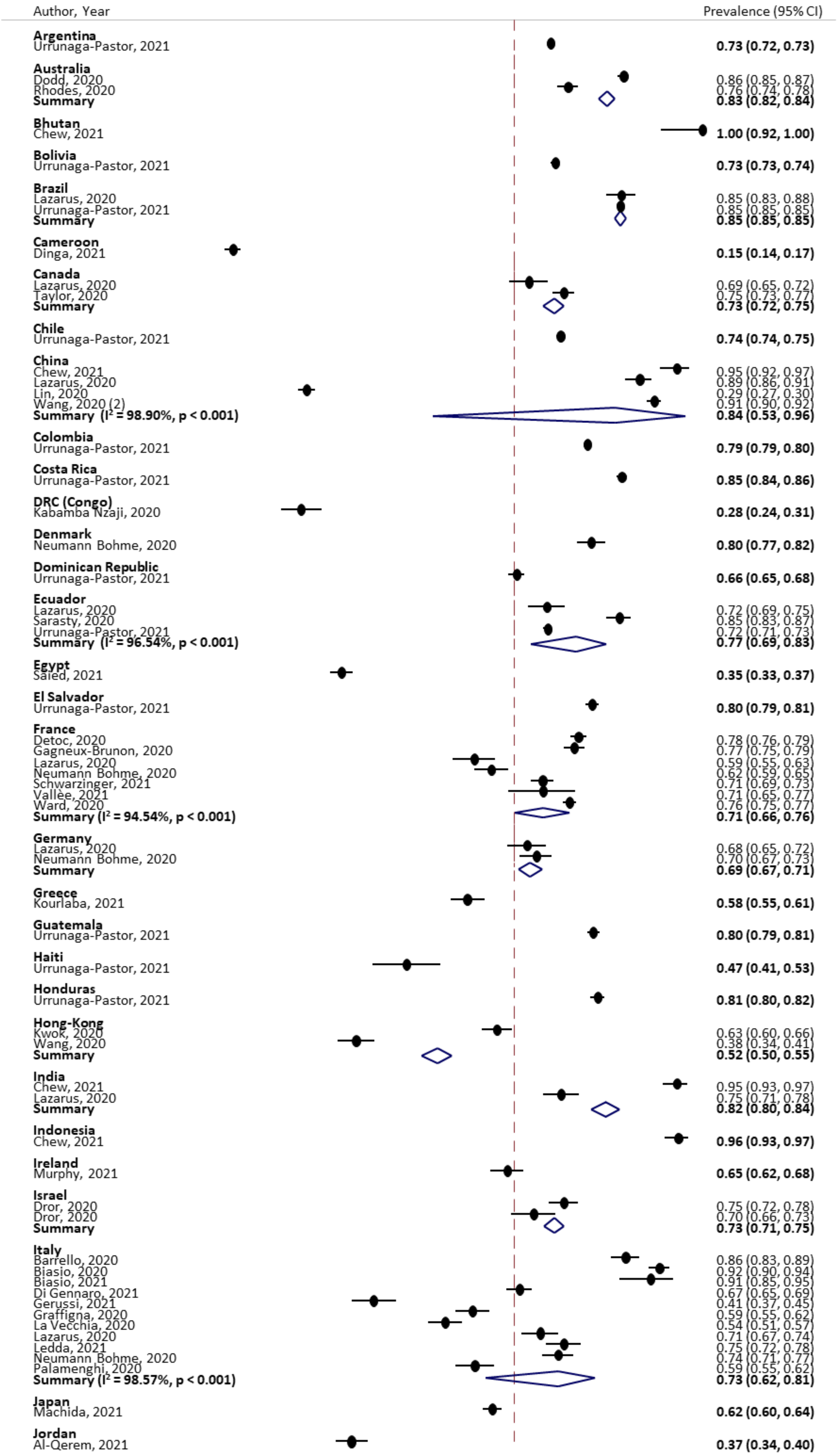

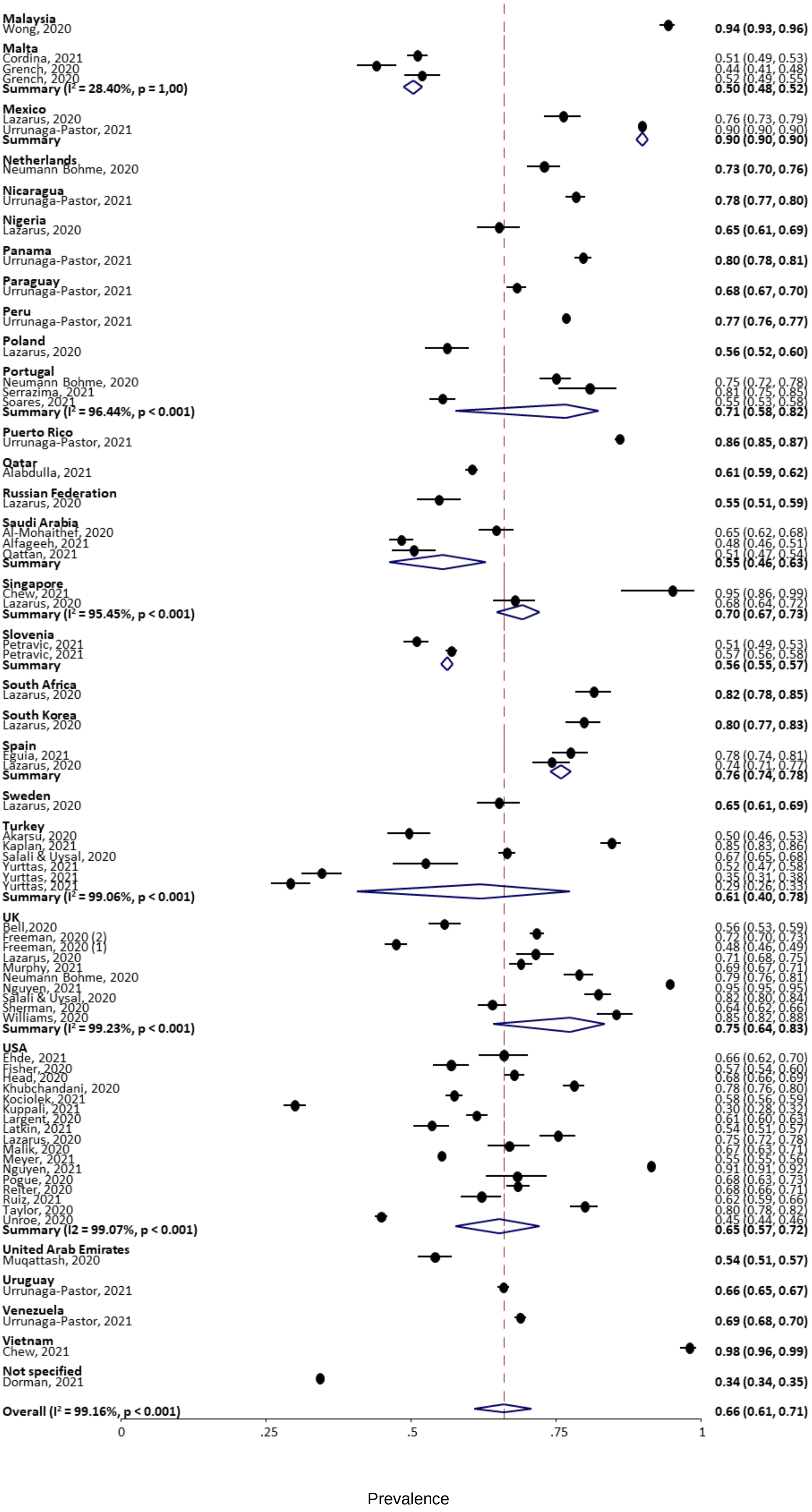

Malaysia
Wong, 2020 0.94 (0.93, 0.96)
Malta
Cordina, 2021 0.51 (0.49, 0.53)
Grench, 2020 0.44 (0.41, 0.48)
Grench, 2020 0.52 (0.49, 0.55)
Summary (I² = 28.40%, p = 1,00) 0.50 (0.48, 0.52)
Mexico
Lazarus, 2020 0.76 (0.73, 0.79)
Urrunaga-Pastor, 2021 0.90 (0.90, 0.90)
Summary 0.90 (0.90, 0.90)
Netherlands
Neumann Bohme, 2020 0.73 (0.70, 0.76)
Nicaragua
Urrunaga-Pastor, 2021 0.78 (0.77, 0.80)
Nigeria
Lazarus, 2020 0.65 (0.61, 0.69)
Panama
Urrunaga-Pastor, 2021 0.80 (0.78, 0.81)
Paraguay
Urrunaga-Pastor, 2021 0.68 (0.67, 0.70)
Peru
Urrunaga-Pastor, 2021 0.77 (0.76, 0.77)
Poland
Lazarus, 2020 0.56 (0.52, 0.60)
Portugal
Neumann Bohme, 2020 0.75 (0.72, 0.78)
Serrazima, 2021 0.81 (0.75, 0.85)
Soares, 2021 0.55 (0.53, 0.58)
Summary (I² = 96.44%, p < 0.001) 0.71 (0.58, 0.82)
Puerto Rico
Urrunaga-Pastor, 2021 0.86 (0.85, 0.87)
Qatar
Alabdulla, 2021 0.61 (0.59, 0.62)
Russian Federation
Lazarus, 2020 0.55 (0.51, 0.59)
Saudi Arabia
Al-Mohaithef, 2020 0.65 (0.62, 0.68)
Alfageeh, 2021 0.48 (0.46, 0.51)
Qattan, 2021 0.51 (0.47, 0.54)
Summary 0.55 (0.46, 0.63)
Singapore
Chew, 2021 0.95 (0.86, 0.99)
Lazarus, 2020 0.68 (0.64, 0.72)
Summary (I² = 95.45%, p < 0.001) 0.70 (0.67, 0.73)
Slovenia
Petravic, 2021 0.51 (0.49, 0.53)
Petravic, 2021 0.57 (0.56, 0.58)
Summary 0.56 (0.55, 0.57)
South Africa
Lazarus, 2020 0.82 (0.78, 0.85)
South Korea
Lazarus, 2020 0.80 (0.77, 0.83)
Spain
Eguia, 2021 0.78 (0.74, 0.81)
Lazarus, 2020 0.74 (0.71, 0.77)
Summary 0.76 (0.74, 0.78)
Sweden
Lazarus, 2020 0.65 (0.61, 0.69)
Turkey
Akarsu, 2020 0.50 (0.46, 0.53)
Kaplan, 2021 0.85 (0.83, 0.86)
Salali & Uysal, 2020 0.67 (0.65, 0.68)
Yurttas, 2021 0.52 (0.47, 0.58)
Yurttas, 2021 0.35 (0.31, 0.38)
Yurttas, 2021 0.29 (0.26, 0.33)
Summary (I² = 99.06%, p < 0.001) 0.61 (0.40, 0.78)
UK
Bell,2020 0.56 (0.53, 0.59)
Freeman, 2020 (2) 0.72 (0.70, 0.73)
Freeman, 2020 (1) 0.48 (0.46, 0.49)
Lazarus, 2020 0.71 (0.68, 0.75)
Murphy, 2021 0.69 (0.67, 0.71)
Neumann Bohme, 2020 0.79 (0.76, 0.81)
Nguyen, 2021 0.95 (0.95, 0.95)
Salali & Uysal, 2020 0.82 (0.80, 0.84)
Sherman, 2020 0.64 (0.62, 0.66)
Williams, 2020 0.85 (0.82, 0.88)
Summary (I² = 99.23%, p < 0.001) 0.75 (0.64, 0.83)
USA
Ehde, 2021 0.66 (0.62, 0.70)
Fisher, 2020 0.57 (0.54, 0.60)
Head, 2020 0.68 (0.66, 0.69)
Khubchandani, 2020 0.78 (0.76, 0.80)
Kociolek, 2021 0.58 (0.56, 0.59)
Kuppali, 2021 0.30 (0.28, 0.32)
Largent, 2020 0.61 (0.60, 0.63)
Latkin, 2021 0.54 (0.51, 0.57)
Lazarus, 2020 0.75 (0.72, 0.78)
Malik, 2020 0.67 (0.63, 0.71)
Meyer, 2021 0.55 (0.55, 0.56)
Nguyen, 2021 0.91 (0.91, 0.92)
Pogue, 2020 0.68 (0.63, 0.73)
Reiter, 2020 0.68 (0.66, 0.71)
Ruiz, 2021 0.62 (0.59, 0.66)
Taylor, 2020 0.80 (0.78, 0.82)
Unroe, 2020 0.45 (0.44, 0.46)
Summary (I2 = 99.07%, p < 0.001) 0.65 (0.57, 0.72)
United Arab Emirates
Muqattash, 2020 0.54 (0.51, 0.57)
Uruguay
Urrunaga-Pastor, 2021 0.66 (0.65, 0.67)
Venezuela
Urrunaga-Pastor, 2021 0.69 (0.68, 0.70)
Vietnam
Chew, 2021 0.98 (0.96, 0.99)
Not specified
Dorman, 2021 0.34 (0.34, 0.35)
Overall (I² = 99.16%, p < 0.001) 0.66 (0.61, 0.71)
0
.25
.5
.75
1
Prevalence

Supplementary Figure S2. Forest plot of meta-analysis proportion (by geographical area).

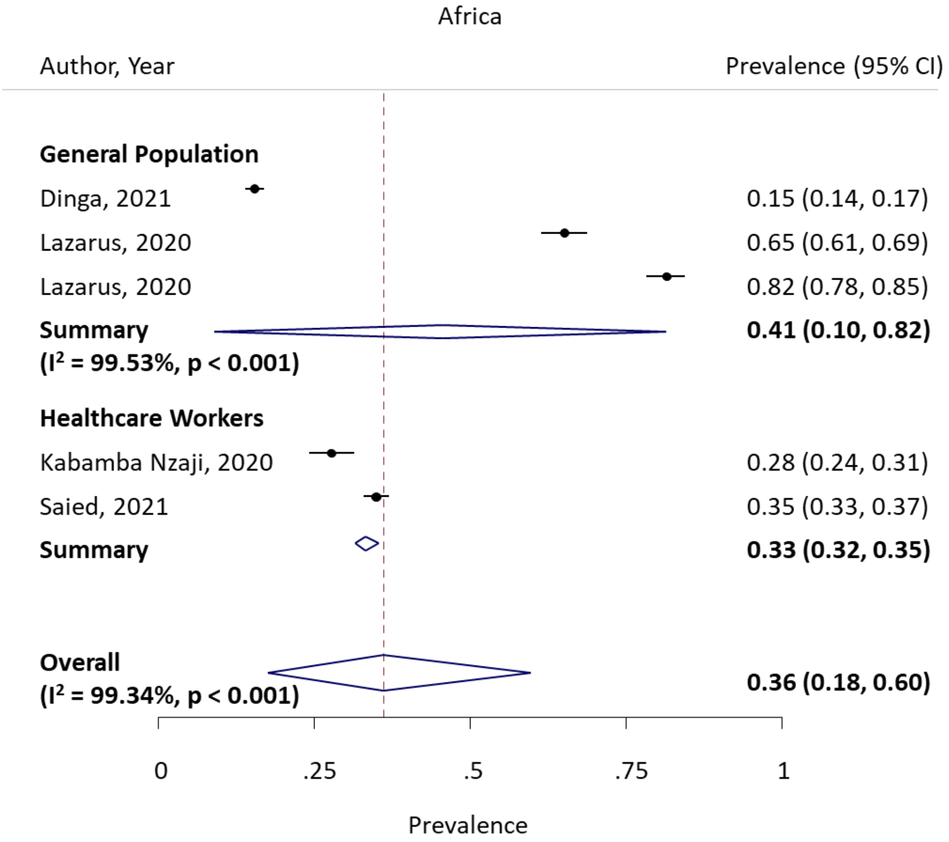

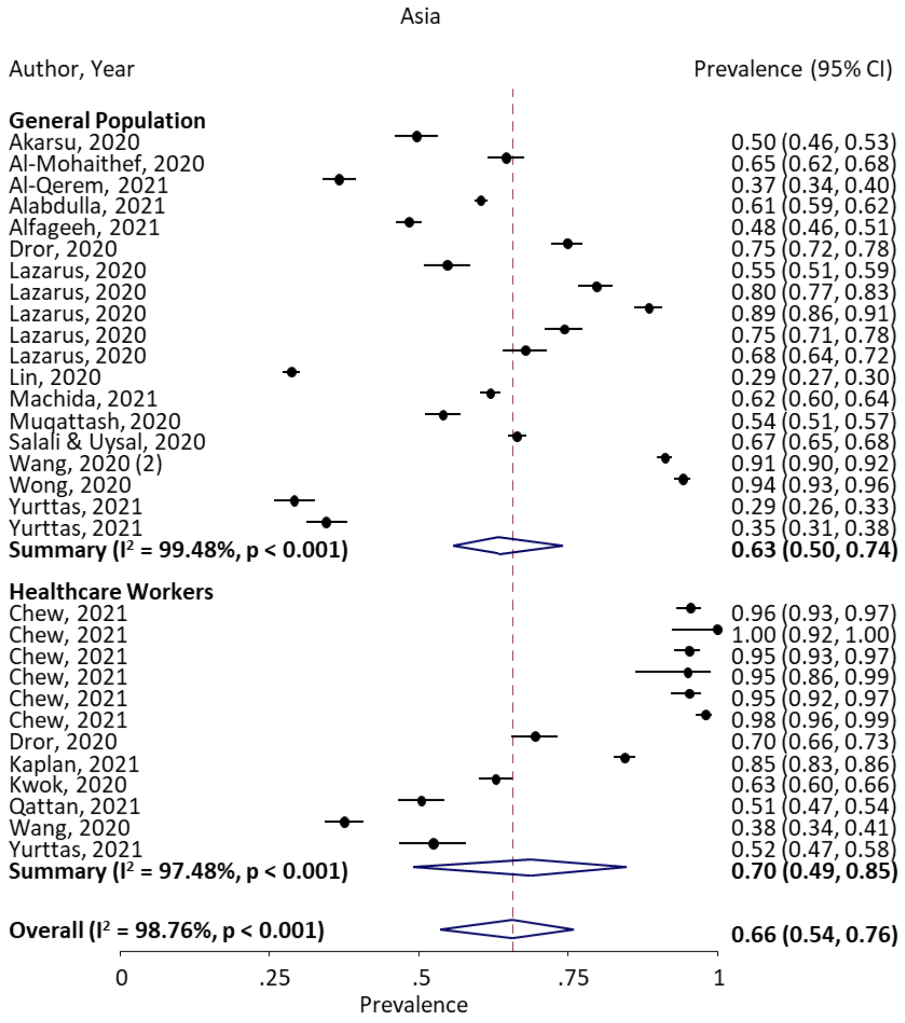

Asia
Author, Year
Prevalence (95% CI)
General Population
Akarsu, 2020 0.50 (0.46, 0.53)
Al-Mohaithef, 2020 0.65 (0.62, 0.68)
Al-Qerem, 2021 0.37 (0.34, 0.40)
Alabdulla, 2021 0.61 (0.59, 0.62)
Alfageeh, 2021 0.48 (0.46, 0.51)
Dror, 2020 0.75 (0.72, 0.78)
Lazarus, 2020 0.55 (0.51, 0.59)
Lazarus, 2020 0.80 (0.77, 0.83)
Lazarus, 2020 0.89 (0.86, 0.91)
Lazarus, 2020 0.75 (0.71, 0.78)
Lazarus, 2020 0.68 (0.64, 0.72)
Lin, 2020 0.29 (0.27, 0.30)
Machida, 2021 0.62 (0.60, 0.64)
Muqattash, 2020 0.54 (0.51, 0.57)
Salali & Uysal, 2020 0.67 (0.65, 0.68)
Wang, 2020 (2) 0.91 (0.90, 0.92)
Wong, 2020 0.94 (0.93, 0.96)
Yurttas, 2021 0.29 (0.26, 0.33)
Yurttas, 2021 0.35 (0.31, 0.38)
Summary (I2 = 99.48%, p < 0.001) 0.63 (0.50, 0.74)
Healthcare Workers
Chew, 2021 0.96 (0.93, 0.97)
Chew, 2021 1.00 (0.92, 1.00)
Chew, 2021 0.95 (0.93, 0.97)
Chew, 2021 0.95 (0.86, 0.99)
Chew, 2021 0.95 (0.92, 0.97)
Chew, 2021 0.98 (0.96, 0.99)
Dror, 2020 0.70 (0.66, 0.73)
Kaplan, 2021 0.85 (0.83, 0.86)
Kwok, 2020 0.63 (0.60, 0.66)
Qattan, 2021 0.51 (0.47, 0.54)
Wang, 2020 0.38 (0.34, 0.41)
Yurttas, 2021 0.52 (0.47, 0.58)
Summary (I2 = 97.48%, p < 0.001) 0.70 (0.49, 0.85)
Overall (I2 = 98.76%, p < 0.001) 0.66 (0.54, 0.76)
0
.25
.5
.75
1
Prevalence

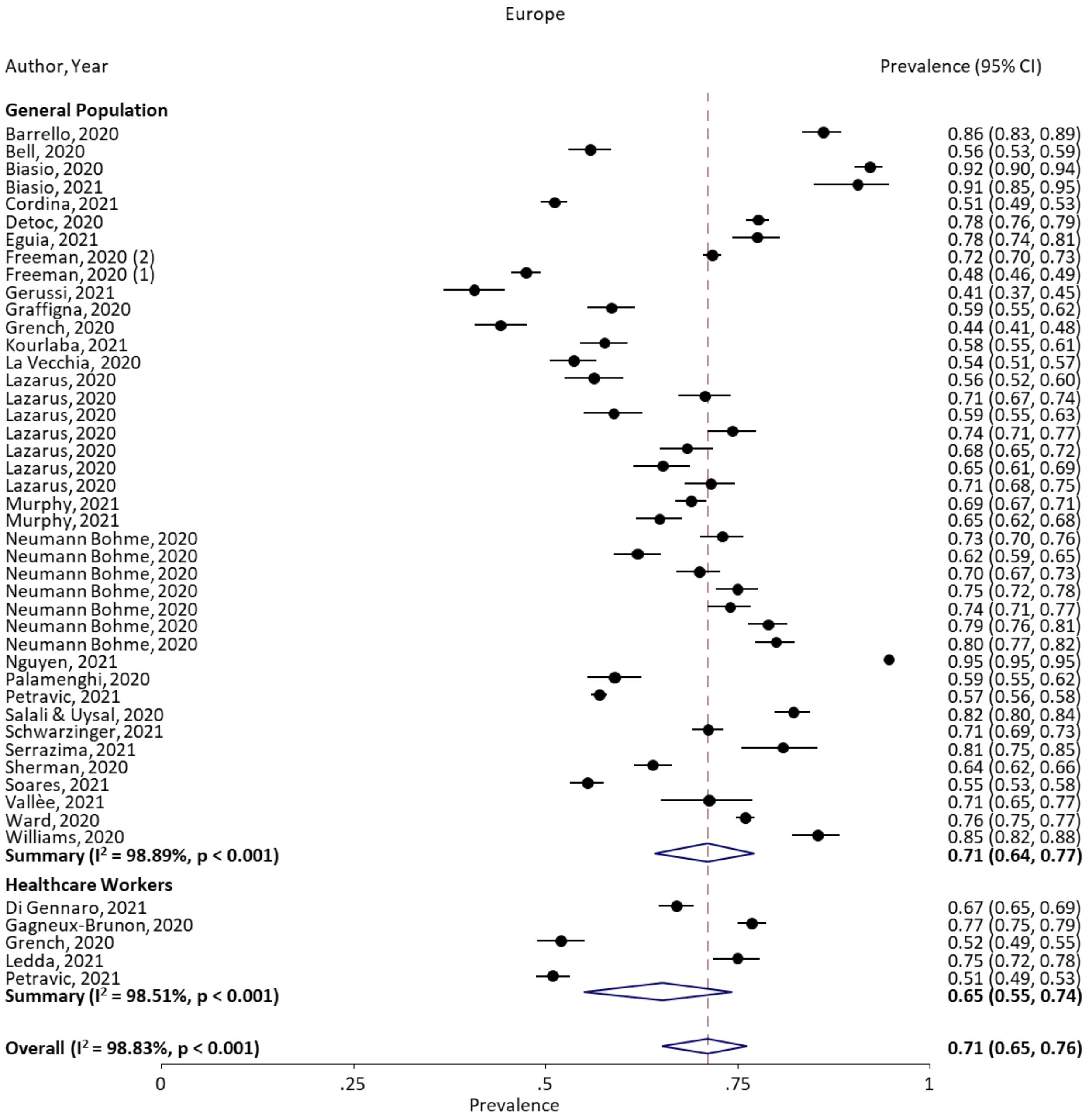
Europe
Author, Year
Prevalence (95% CI)
General Population
Barrello, 2020 0.86 (0.83, 0.89)
Bell, 2020 0.56 (0.53, 0.59)
Biasio, 2020 0.92 (0.90, 0.94)
Biasio, 2021 0.91 (0.85, 0.95)
Cordina, 2021 0.51 (0.49, 0.53)
Detoc, 2020 0.78 (0.76, 0.79)
Eguia, 2021 0.78 (0.74, 0.81)
Freeman, 2020 (2) 0.72 (0.70, 0.73)
Freeman, 2020 (1) 0.48 (0.46, 0.49)
Gerussi, 2021 0.41 (0.37, 0.45)
Graffigna, 2020 0.59 (0.55, 0.62)
Grench, 2020 0.44 (0.41, 0.48)
Kourlaba, 2021 0.58 (0.55, 0.61)
La Vecchia, 2020 0.54 (0.51, 0.57)
Lazarus, 2020 0.56 (0.52, 0.60)
Lazarus, 2020 0.71 (0.67, 0.74)
Lazarus, 2020 0.59 (0.55, 0.63)
Lazarus, 2020 0.74 (0.71, 0.77)
Lazarus, 2020 0.68 (0.65, 0.72)
Lazarus, 2020 0.65 (0.61, 0.69)
Lazarus, 2020 0.71 (0.68, 0.75)
Murphy, 2021 0.69 (0.67, 0.71)
Murphy, 2021 0.65 (0.62, 0.68)
Neumann Bohme, 2020 0.73 (0.70, 0.76)
Neumann Bohme, 2020 0.62 (0.59, 0.65)
Neumann Bohme, 2020 0.70 (0.67, 0.73)
Neumann Bohme, 2020 0.75 (0.72, 0.78)
Neumann Bohme, 2020 0.74 (0.71, 0.77)
Neumann Bohme, 2020 0.79 (0.76, 0.81)
Neumann Bohme, 2020 0.80 (0.77, 0.82)
Nguyen, 2021 0.95 (0.95, 0.95)
Palamenghi, 2020 0.59 (0.55, 0.62)
Petravic, 2021 0.57 (0.56, 0.58)
Salali & Uysal, 2020 0.82 (0.80, 0.84)
Schwarzinger, 2021 0.71 (0.69, 0.73)
Serrazima, 2021 0.81 (0.75, 0.85)
Sherman, 2020 0.64 (0.62, 0.66)
Soares, 2021 0.55 (0.53, 0.58)
Vallèe, 2021 0.71 (0.65, 0.77)
Ward, 2020 0.76 (0.75, 0.77)
Williams, 2020 0.85 (0.82, 0.88)
Summary (I2 = 98.89%, p < 0.001) 0.71 (0.64, 0.77)
Healthcare Workers
Di Gennaro, 2021 0.67 (0.65, 0.69)
Gagneux-Brunon, 2020 0.77 (0.75, 0.79)
Grench, 2020 0.52 (0.49, 0.55)
Ledda, 2021 0.75 (0.72, 0.78)
Petravic, 2021 0.51 (0.49, 0.53)
Summary (I2 = 98.51%, p < 0.001) 0.65 (0.55, 0.74)
Overall (I2 = 98.83%, p < 0.001) 0.71 (0.65, 0.76)
0
.25
.5
.75
1
Prevalence

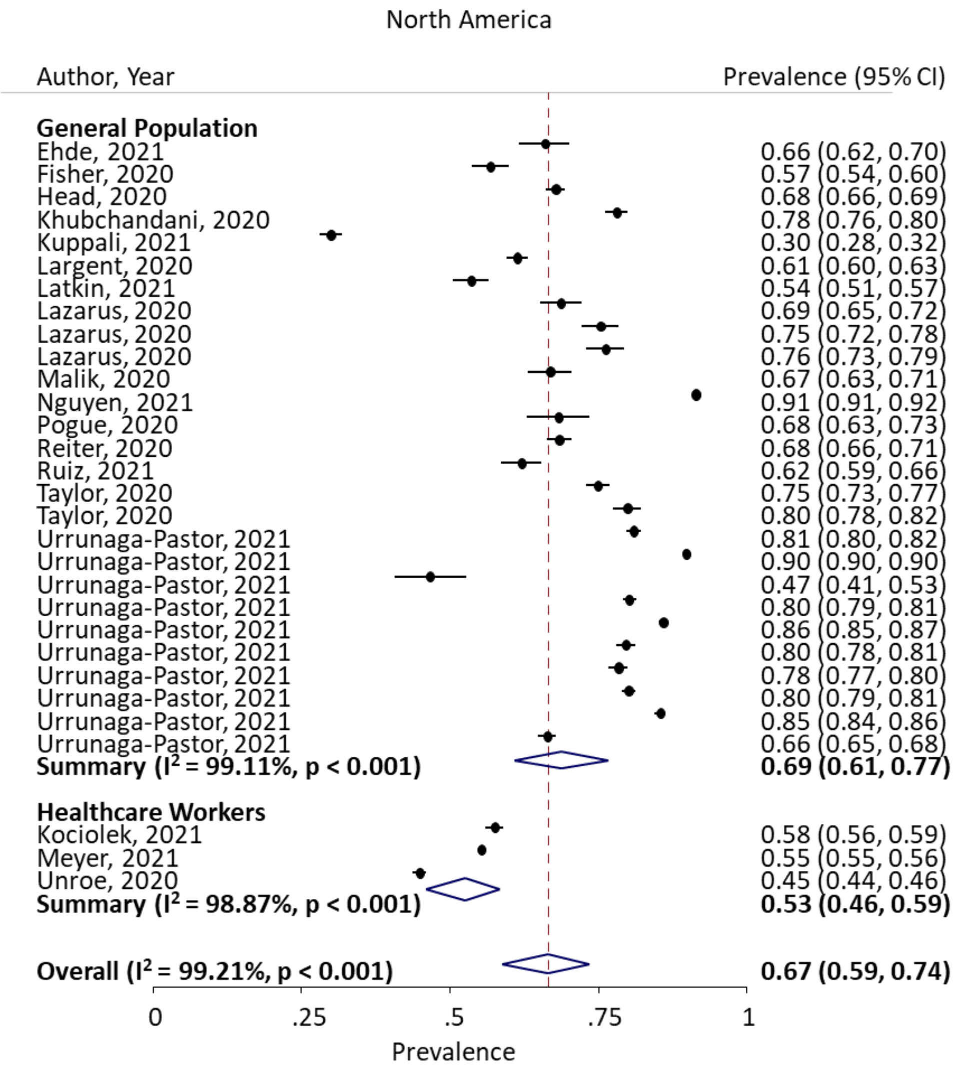
North America
Author, Year
Prevalence (95% CI)
General Population
Ehde, 2021 0.66 (0.62, 0.70)
Fisher, 2020 0.57 (0.54, 0.60)
Head, 2020 0.68 (0.66, 0.69)
Khubchandani, 2020 0.78 (0.76, 0.80)
Kuppali, 2021 0.30 (0.28, 0.32)
Largent, 2020 0.61 (0.60, 0.63)
Latkin, 2021 0.54 (0.51, 0.57)
Lazarus, 2020 0.69 (0.65, 0.72)
Lazarus, 2020 0.75 (0.72, 0.78)
Lazarus, 2020 0.76 (0.73, 0.79)
Malik, 2020 0.67 (0.63, 0.71)
Nguyen, 2021 0.91 (0.91, 0.92)
Pogue, 2020 0.68 (0.63, 0.73)
Reiter, 2020 0.68 (0.66, 0.71)
Ruiz, 2021 0.62 (0.59, 0.66)
Taylor, 2020 0.75 (0.73, 0.77)
Taylor, 2020 0.80 (0.78, 0.82)
Urrunaga-Pastor, 2021 0.81 (0.80, 0.82)
Urrunaga-Pastor, 2021 0.90 (0.90, 0.90)
Urrunaga-Pastor, 2021 0.47 (0.41, 0.53)
Urrunaga-Pastor, 2021 0.80 (0.79, 0.81)
Urrunaga-Pastor, 2021 0.86 (0.85, 0.87)
Urrunaga-Pastor, 2021 0.80 (0.78, 0.81)
Urrunaga-Pastor, 2021 0.78 (0.77, 0.80)
Urrunaga-Pastor, 2021 0.80 (0.79, 0.81)
Urrunaga-Pastor, 2021 0.85 (0.84, 0.86)
Urrunaga-Pastor, 2021 0.66 (0.65, 0.68)
Summary (I^2 = 99.11%, p < 0.001) 0.69 (0.61, 0.77)
Healthcare Workers
Kociolek, 2021 0.58 (0.56, 0.59)
Meyer, 2021 0.55 (0.55, 0.56)
Unroe, 2020 0.45 (0.44, 0.46)
Summary (I^2 = 98.87%, p < 0.001) 0.53 (0.46, 0.59)
Overall (I^2 = 99.21%, p < 0.001) 0.67 (0.59, 0.74)
0
.25
.5
.75
1
Prevalence

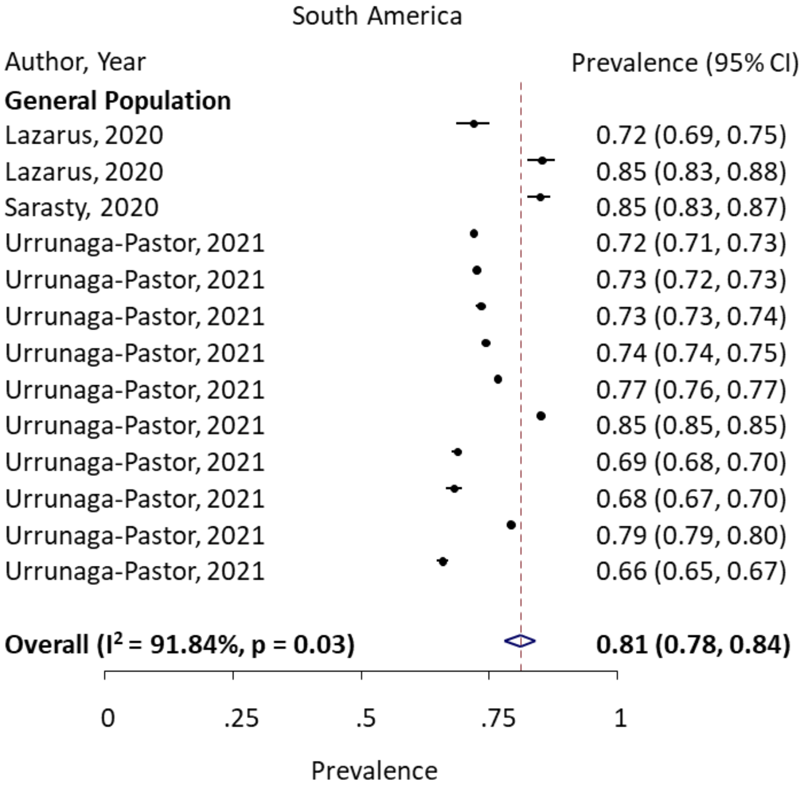
South America
Author, Year
Prevalence (95% CI)
General Population
Lazarus, 2020 0.72 (0.69, 0.75)
Lazarus, 2020 0.85 (0.83, 0.88)
Sarasty, 2020 0.85 (0.83, 0.87)
Urrunaga-Pastor, 2021 0.72 (0.71, 0.73)
Urrunaga-Pastor, 2021 0.73 (0.72, 0.73)
Urrunaga-Pastor, 2021 0.73 (0.73, 0.74)
Urrunaga-Pastor, 2021 0.74 (0.74, 0.75)
Urrunaga-Pastor, 2021 0.77 (0.76, 0.77)
Urrunaga-Pastor, 2021 0.85 (0.85, 0.85)
Urrunaga-Pastor, 2021 0.69 (0.68, 0.70)
Urrunaga-Pastor, 2021 0.68 (0.67, 0.70)
Urrunaga-Pastor, 2021 0.79 (0.79, 0.80)
Urrunaga-Pastor, 2021 0.66 (0.65, 0.67)
Overall (I^2 = 91.84%, p = 0.03) 0.81 (0.78, 0.84)
0
.25
.5
.75
1
Prevalence

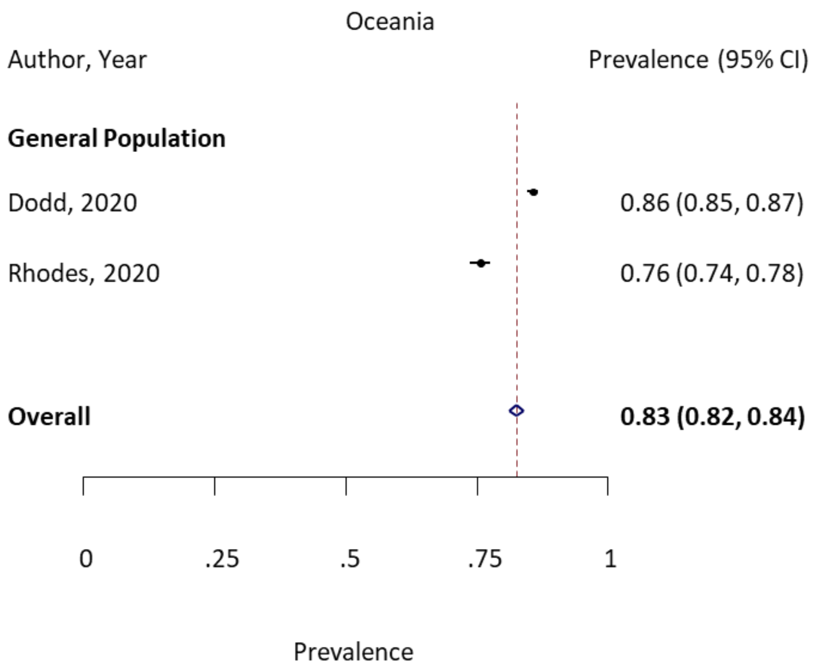

Oceania
Author, Year
Prevalence (95% CI)
General Population
Dodd, 2020
0.86 (0.85, 0.87)
Rhodes, 2020
0.76 (0.74, 0.78)
Overall
0.83 (0.82, 0.84)
0
.25
.5
.75
1
Prevalence
